# Lung cancer targeting efficiency of Silibinin loaded Poly Caprolactone /Pluronic F68 Inhalable nanoparticles: *In vitro* and *In vivo* study

**DOI:** 10.1371/journal.pone.0267257

**Published:** 2022-05-13

**Authors:** Priya Patel, Mihir Raval, Aneka Manvar, Vishal Airao, Vaibhav Bhatt, Pranav Shah

**Affiliations:** 1 Department of Pharmaceutical Sciences, Saurashtra University, Rajkot, Gujarat, India; 2 Department of Pharmaceutical Sciences, Sardar Patel University, Vallabh Vidyanagar, Gujarat, India; 3 School of Applied sciences and Technology, Gujarat Technological University, Ahmedabad, Gujarat, India; 4 Maliba Pharmacy College, Uka Tarsadia University, Tarsadi, Gujarat, India; ISF College of Pharmacy, Moga, Punjab, India, INDIA

## Abstract

Silibinin (SB) is shown to have an anticancer properties. However, its clinical therapeutic effects have been restricted due to its low water solubility and poor absorption after oral administration. The aim of this study was to develop SB-loaded PCL/Pluronic F68 nanoparticles for pulmonary delivery in the treatment of lung cancer. A modified solvent displacement process was used to make nanoparticles, which were then lyophilized to make inhalation powder, Nanoparticles were characterized with DSC, FTIR,SEM and *In vitro* release study. Further, a validated HPLC method was developed to investigate the Biodistribution study, pharmacokinetic parameters. Poly Caprolactone PCL / Pluronic F68 NPs showed the sustained release effect up to 48 h with an emitted (Mass median Aerodynamic diameter)MMAD and (Geometric size distribution)GSD were found to be 4.235 ±0.124 and 1.958±1.23 respectively. More specifically, the SB Loaded PCL/Pluronic F 68 NPs demonstrated long circulation and successful lung tumor-targeting potential due to their cancer-targeting capabilities. SB Loaded PCL/Pluronic F68 NPs significantly inhibited tumour growth in lung cancer-induced rats after inhalable administration. In a pharmacokinetics study, PCL/ Pluronic F68 NPs substantially improved SB bioavailability, with a more than 4-fold rise in AUC when compared to IV administration. These findings indicate that SB-loaded PCL/PluronicF68 nanoparticles may be a successful lung cancer therapy delivery system.

## Introduction

Lung cancer is a lethal, violent, and progressive disease with limited treatment options and a poor prognosis in the early stages. Despite recent advancements in care for other cancers, the 5-year survival rate of patients with lung cancer is still just 16% at all stages [1]. The main drawbacks of current treatments are required extremely large doses, damage to healthy tissue cells as well as difficult to removing cancer tissues and multi drug resistance [2]. Multidrug resistance (MDR) to anticancer agents remains a significant obstacle to the effective treatment of cancer. Given all of these disadvantages as well as the mortality rates from lung cancer, a therapeutic strategy that can improve efficacy is urgently needed. Consequently, successful therapies to overcome MDR against invasive lung cancer and particularly highly metastatic diseases remain an important priority [3].

Through reducing their efflux from P-glycoprotein-mediated cells, nanoparticles can reduce the multidrug resistance (MDR) of many anticancer drugs, including Silibinin [4]. Nanoparticles are distributed across the body based on a variety of factors, including their small size, which contributes to longer circulation periods, and their ability to take advantage of tumour properties. Traditional chemotherapy is usually limited by drug toxicity to normal tissues, short half-life circulation in plasma, reduced aqueous solubility, and therapeutic efficacy. Nanoparticles and their use in drug delivery are a much more efficient method of cancer treatment than conventional chemotherapy [5].

Silibinin, a chemically specified plant-based compound, is the most biologically active component of the silymarin complex, which is made up of two diastereoisomers and is derived from the seeds of the milk thistle plant (silybin A and silybin B) [6, 7]. Silibinin has also been shown to have an inhibitory effect on cancers such as liver cancer, prostate cancer, colon cancer, breast cancer and skin cancer in recent studies [8, 9]. Nevertheless, its efficacy was severely limited due to low aqueous solubility (0.092 mg / ml) and poor oral bioavailability, 23–47% after oral administration [10, 11]. Large doses of SB are required for attaining therapeutic plasma levels. To meet this need, researchers around the globe have developed a variety of delivery systems to enhance its solubility and thus bioavailability. It is therefore important to explore new formulations to address the above-mentioned limitations.

Among, for this purpose the formulation of nanoparticles holds the greatest promise. The nanoparticles exhibited benefits over others, such as greater stability during storage. As a result, a colloidal device like this will extravasate solid tumours to inflamed or contaminated capillary endothelium defects [4, 5].

There have been various advances in drug delivery nowadays; however, nano-systems based on polymer tend to be growing in popularity. Polymeric nanoparticles (NP) made of polymers of 10–1000 nm size, which are small enough to penetrate easily through intercellular tumor gaps, particularly in tumors suffering from angiogenesis [12]. The intercellular gaps of cancer cells widen as blood vessel size increases, allowing a drug delivery system to be passively targeted, increasing drug selectivity and minimising adverse effects [13]. Poly(ε-caprolactone) (PCL) nanoparticles (NP) offer many possibilities for drug transport because of their good physicochemical properties, biocompatibility and their multi-functionality [14, 15]. In fact, a number of PCL-nanoformulations have been produced (micelles, hydrogels, scaffolds, fibres, films, and microspheres) [16] that have successfully loaded a variety of antitumor drugs in both in vitro and in vivo cancer models [17]. However, the slow degradation of PCL-based particles enables the prolonged release of the drug [18]. Lack of toxicity and high permeability have already been widely used for PCL medicinal applications [19]. Pluronic F68 is a main hydroxyl group terminating difunctional copolymer block surfactant. It is soluble in both water and organic solvents. Poloxamers and nonionic poloxamine surfactants are used in a number of biomedical applications, including drug delivery and medical imaging, as well as the prevention and treatment of vascular disease and disorders [20]. Pluronic F68 was incorporated in the present study as a pore-forming agent and drug-releasing enhancer in PCL.

Pluronic block copolymers have also been demonstrated to interfere with MDR tumours, leading them to become significantly more receptive to anticancer treatments [21, 22]. Pluronics’ biological activity is defined by its ability to bind to membranes, followed by cell translocation, and influence cellular activities like mitochondrial respiration, ATP generation, apoptotic signal transmission, and gene expression. Pluronics, as a result, allows MDR tumours to become significantly more receptive to various anticancer agents, including silibinin, improves drug delivery through blood-brain and intestinal barriers, and induces transcriptional activation of gene expression in vitro and in vivo [22, 23]. Furthermore, recent studies showed that Pluronic F68 is both a potent P-gp and CYP inhibitor [24].

By taking advantage of the wide surface area, thin alveolar epithelium, permeable membrane, and extensive vasculature, drug delivery via the inhalation route of administration has the capacity to enable a high degree of local absorption [25]. Therefore, the use of local passive administration to the tumor site of an ideal inhalable lung cancer therapy facilitates optimum therapeutic drug concentration while retaining lower adverse side effects associated with systemic administration [26]. Inhalation administration provides a non-invasive means of circumventing first-pass metabolism, reducing the therapeutic dosage and frequency of administration, and delivering drugs directly to their location of action with increased concentrations of local drugs, thus reducing the potential for systemic toxicity [27]. A safe and effective drug delivery system that releases drug in a sustained manner is desirable to limit exposure to normal tissues while delivering the active chemotherapeutic to the cancer cells [28].

The development of the formulation process involves a vital understanding of the effect of the factors in the formulation. The traditional one variable at a time (OVAT) formulation optimization technique assesses the effect of individual variables on responses [29]. As a result, systematic design of experiments (DoE) is a method for visualising response variance as a function of factor. A central composite design (CCD) is a type of factorial design in which the ‘star points’ are placed at a distance a from the centre and have a value equal to the square root of the number of variables. It may also have circumscribed, inscribed, or faced design based on the position of ‘star points’ [30].

The present research work was aimed to develop SB loaded PCL/Pluronic F68 NPs to improve their bioavailability and anti-cancer activity with sustained release of drug by Inhalation approach. The effect of independent variables on dependent variables and their responses was investigated using a central composite design. Parameters such as particle size, PDI, zeta potential, Entrapment Efficiency, drug content, *In vitro* drug release study along with the *In vitro* anticancer activity and *In vivo* study were evaluated for the optimized formulation. We are investigating the hypothesis that a novel PCL / Pluronic F68 nanoparticles charged with SB would achieve improved therapeutic results in the lineage of human lung cancer to SB.

Thus, in this research work. We are researching on a nanoparticulate drug delivery method that will improve oral bioavailability by delivering the drug in a sustained manner to the deep region of the lungs and retaining it for a longer period of time.

## Materials and methods

Silibinin (SB), Poly Vinyl Alcohol (Mw 30,000–70,000 kDa), Polycaprolactone (PCL), Inhalable grade lactose, octoate stannous was purchased from Sigma Aldrich Pvt Ltd., Mumbai, India Pluronic F68 was purchased from BASF, Mumbai, India as surfactants. All of the other solvents utilized in the experiment were of analytical grade. In all of the studies, double distilled water was used.

### Synthesis of PCL/Pluronic F68 polymer

Bulk polymerization was used to make the PCL/Pluronic F68 compound [31]. Briefly, the terminal hydroxyl groups in Pluronic F68 molecules were capped with acetyl render them inactive and prevent them from participating in the polymerization reaction of ε-caprolactone. Before polymerization, the acetyl-capped F68 was dissolved in e-caprolactone monomer, allowing F68 to be incorporated into PCL matrixes as a molecular dispersion rather than forming a copolymer. The polymerization was carried out with 0.04% octoate stannous as a catalyst at 140° C for 24 hours under high vacuum.

### Formulation of PCL/Pluronic F68 nanoparticles

A modified solvent evaporation method was used to prepared SB loaded PCL / Pluronic F68 nanoparticles [32]. Briefly, the required quantity of PCL / Pluronic F68 polymer and 50 mg of SB in 50 mL of acetone were dissolved. Under magnetic stirring, the organic phase was poured into 25 ml of double-distilled water containing PVA. The resulting solution was continuously stirred to completely evaporate the organic solvent at room temperature for 6h. The resulting nanoparticulate suspension was kept for ultrasonication in a Probe sonicator (Frontline Electronics And Machinery Pvt Ltd., Ahmedabad, India) and centrifuged (Remi Laboratory Instruments, Mumbai, India) at 15,000 rpm for 30 min and washed twice with double-distilled water [32]. Same way SB Loaded PCL Nanoparticles were prepared by a modified solvent evaporation method using PCL as a polymer.

### Freeze drying of PCL/Pluronic F68 nanoparticles

The Free Zone 2.5 Litre Benchtop Freeze Dryer was used to freeze-dried of SB loaded PCL / Pluronic F68 Nanoparticles (Labconco, USA). 2 mL formulations were put into semi-stoppered glass vials with slotted rubber closures, then diluted with 2 mL of mannitol solution (25% w/w) as a cryoprotectant. The sample was frozen at -50°C in the lyophilizer chamber for 48 hours, then dried at -32°C and 150 mtorr for 24h, subsequently dried at 20°C and 50 mtorr for 6 h. After freeze drying, the final concentration of SB loaded PCL / Pluronic F68 Nanoparticles was 100 mg/g [33]. The similar procedure was used to dried SB loaded PCL nanoparticles.

### Optimization of PCL/Pluronic F68 nanoparticles formulation using central composite design

For estimating second-order response surfaces, the central composite design (CCD), an augmented factorial design, is widely used. Six replicate runs at the centre stage, coded 0 were carried out to assess the experimental error and verify the overall curvature effect. To make the geometry rotatable, the distance between the axial points and the centre point was determined using = 2k/4 (k is the number of independent variables, which in this study was 1.68). The design consisted of three groups of design points for each of the two factors: two factorial level design points fixed at +1 and -1; a centre level design point fixed at 0; and two star level design points fixed at + and -; the value of for a two factor analysis is 1.414. Design-Expert version 11.0.0.0 was used for all statistical analyses with Central composite design (Stat-Ease, Inc., Minneapolis, Minnesota, USA). Table 1 shows the variables and their levels. As a result, 20 runs were carried out in the current study using central points [34]. To eradicate potential sources of bias, all experiments were conducted in a randomised fashion. As shown in Eq 1, a statistical mathematical model term was used to analyze the responses.

$$\text{Y}={\text{b}}_{0}+{\text{b}}_{1}{\text{X}}_{1}+{\text{b}}_{2}{\text{X}}_{2}+{\text{b}}_{3}{\text{X}}_{3}+{\text{b}}_{12}{\text{X}}_{1}{\text{X}}_{2}+{\text{b}}_{13}{\text{X}}_{1}{\text{X}}_{3}+{\text{b}}_{23}{\text{X}}_{2}{\text{X}}_{3}+{\text{b}}_{11}{\text{X}}_{1}{}^{2}+{\text{b}}_{22}{\text{X}}_{2}{}^{2}+{\text{b}}_{33}{\text{X}}_{3}{}^{2}$$ (1)

Where, Y is measured response, b_0_ is the constant, b_1_, b_2_ and b_3_ are coefficient for the factor, X_1_, X_2_, X_3_, b_12_, b_13_ and b_23_ are coefficient of interaction, b_11_, b_22_ and b_33_ are coefficient of quadratic term.

**Table 1:** Particle size and entrapment efficiency of design batches.

Formulation code	Actual value			Particle size (nm)*	% Entrapment efficiency*
	X1	X2	X3		
B1	50	0.5	5	101±1.12	54.04±1.13
B2	150	0.5	5	198±2.21	60.51±1.87
B3	50	1.5	5	232±2.11	68.76±0.64
B4	150	1.5	5	368±1.05	76.75±1.25
B5	50	0.5	15	217±2.04	67.45±2.10
B6	150	0.5	15	390±3.25	73.51±2.25
B7	50	1.5	15	169±2.24	77.65±3.11
B8	150	1.5	15	204±2.08	71.08±2.39
B9	160	1	10	200±3.10	69.79±1.21
B10	180	1	10	397±3.19	67.76±2.32
B11	100	0.8	10	127±1.08	52.51±1.31
B12	100	1.2	10	108±3.21	70.29±2.40
B13	100	1	16	154±2.22	81.08±1.28
B14	100	1	18	126±1.24	74.07±2.19
B15	100	1	10	145±2.27	71.81±1.32
B16	100	1	10	249±1.17	59.64±2.29
B17	100	1	10	115±2.31	68.39±1.18
B18	100	1	10	130±1.41	67.97±2.22
B19	100	1	10	109±1.32	58.39±1.21
B20	100	1	10	294±2.29	61.96±2.26

X_1_ = Drug: Polymer ratio (mg), X_2_ = Surfactant concentration (%), X_3_ = Sonication time (min)

*The results are in triplicate (n = 3)

All the batches were prepared according to the experimental design shown in Table 2 summarizes an account of the 20 runs studies, their factor combinations and the translation of the coded levels to the experimental units employed during the study.

**Table 2:** Summary of ANOVA.

**Particle Size**					
**Source**	**Sum of Square**	**Df**	**Mean Square**	**F value**	**P value**
Model	7845	14	8745.2	14.28	0.0014
X_1_	8547	1	8547.32	48.54	0.0147
X_2_	4152.2	1	4152.2	16.52	0.0214
X_3_	1025.32	1	1025.32	1.25	0.1025
X_12_	2145	1	2145	2.65	0.1895
X_23_	1147	1	1147	4.87	0.2654
X_31_	758.5	1	758.5	32.54	0.3214
X_1_^2^	879.5	1	879.5	28.65	0.0254
X_2_^2^	123.5	1	123.5	1.25	0.6541
X_3_^2^	89.65	1	89.65	1.45	0.8745
Residual	18,785	15	2145.2		
Lack of Fit	14,775	10	1054.2	1.58	0.2145
Pure Error	1745.21	5	875.6		
**% Entrapment Efficiency**					
**Source**	**Sum of Square**	**Df**	**Mean Square**	**F value**	**P value**
Model	5854	14	1024.12	12.24	0.0021
X_1_	214.52	1	214.52	2.251	0.0147
X_2_	54.21	1	54.21	0.021	0.0235
X_3_	25.21	1	25.21	1.854	0.0321
X_12_	41.25	1	41.25	2.321	0.8741
X_23_	9.85	1	9.85	3.214	0.6548
X_31_	1.25	1	1.25	0.895	0.0147
X_1_^2^	87.54	1	87.54	0.654	0.0245
X_2_^2^	102.25	1	102.25	1.457	0.0478
X_3_^2^	78.54	1	78.54	1.214	0.0548
Residual	98.54	15			
Lack of Fit	52.21	10	6.87	1.87	0.3547
Pure Error	25.10	5			

### Data optimization and validation of response surface methodology

Design-Expert software was used to perform various RSM computations for the current optimization analysis (version 11.0.0.0). Using a multiple linear regression analysis technique, a polynomial model with interaction and quadratic terms was created for all of the response variables. In terms of statistical coefficients and R^2^ values, the models were assessed. Design-Expert software was used to construct 3-D surface plots and 2-D contour plots. To verify RSM, one optimized formulation was chosen as a checkpoint. In order to determine the model’s validity, the percentage relative error for each response was determined using Eq 2 [35].


$$\%\;\text{Relative}\;\text{Error}=\frac{Predicted\;Value-Experimental\;Value}{Predicted\;Value}\;x\;100$$ (2)

### Evaluation of PCL/Pluronic F68 nanoparticles

#### Particle size, polydispersity index, and ζ-potential

Zetasizer-Nano ZS (ZEN3600 Malvern Zetasizer Nanoseries Nano-ZS, UK)was used to determine the particle size, Polydipsersity index (PDI) and Zeta potential of the particles by light scattering method. Prior to measurement, each sample was sufficiently diluted with double distilled water, and three measurements were taken for each sample.

#### % entrapment efficiency

The amount of unentrapped SB in the aqueous surfactant solution was used to calculate the percent EE. Centrifugation was used to isolate the aqueous medium. In Eppendorf tubes, 2.0 mL of Nanoparticulate dispersion was placed and centrifuged at 10,000 rpm for 30 minutes at 4 ± 1°C (Remi Instruments Pvt. Ltd., Mumbai, India). For SB quantification, the supernatant was diluted adequately and analyzed using HPLC. The concentration of SB was determined from the calibration curve and the entrapment efficiency (%) was calculated using the following Eq 3

$$\%\;Entrapment\;Efficicecny=\frac{\text{Total}\;\text{amount}\;\text{of}\;\text{Drug}-\text{Amount}\;\text{of}\;\text{Free}\;\text{Drug}}{\text{Total}\;\text{amount}\;\text{of}\;\text{Drug}}\;X\;100$$ (3)

#### High performance liquid chromatography

The HPLC system (Shimadzu Company, Japan) was used to assess drug concentration in the prepared sample, which included a thermostated CTO-20AC column oven and SIL20AC autosampler, as well as an SPD-M 20A PDA detector. Shimadzu Corporation’s Lab solution programme (version 5.53 SP3C) was used for data collection and processing. An Agilent C18 column (4.6 mm 250 mm, 5 m; Thermo ScientificTM Hypersil GOLDTM, MA, USA) was used for chromatographic separation. The injection volume was set at 20 μL. The 90:10 (v/v) methanol and water mobile phase for the chromatographic separation was filtered before being utilised isocratically at room temperature at a flow rate of 1 mL/min, and SB was detected at 288 nm [36].

#### Fourier Transform Infrared spectroscopy (FTIR)

FTIR Spectroscopy (CARY 630, Agilent technologies, USA) was used for recording the IR Spectra of the samples such as SB, PCL/Pluronic F68, SB loaded PCL/Pluronic F68 Nanoparticles. The spectra were obtained using the lab solution software and recorded using the ATR technique (Diamond ATR Crystal, Agilent technologies, USA). All of the spectra were taken at a resolution of 4 cm-1 in the range of 400–4000 cm-1.

#### Differential Scanning Calorimetry (DSC)

DSC analysis of the samples was performed on a previously calibrated DSC 60 (Shimadzu, Tokyo, Japan) for temperature and heat flow precisely using Indium. SB, PCL/Pluronic F68, and SB loaded PCL/Pluronic F68 Nanoparticles DSC spectra were collected. Accurately weighted samples (3 mg) were put in hermetically sealed aluminium pans and heated at a scanning speed of 50 °C/min while purging with nitrogen at a rate of 10 mL/min through the melting temperature range. Endotherms were measured from 40 to 300°C against a reference of a sealed aluminium empty crucible.

#### Scanning Electron Microscopy (SEM)

SEM was used to analyze the Shape and surface morphology (JEOL-JSM-6380LVERDA, USA). Using a vacuum sputter coater, the produced freeze-dried Nanoparticle formulations were coated with a thin layer of gold palladium metal (JFC-1100 fine coat ion sputter, Jeol, Tokyo, Japan). After that, the coated samples were scanned and imaged. At a 10 kV acceleration voltage, photomicrographs were taken.

#### *In vitro* drug release study

Modified dialysis diffusion techniques were used to determine the drug release characteristics from SB loaded PCL/Pluronic F68 Nanoparticles. A dialysis membrane with a pore size of 2.4 nm and MWCO 12 K– 14 K Da was utilised (Himedia Pvt. Ltd., Mumbai). Dialysis membranes were activated by washing with double distilled water for 24 hours and then equilibrated with Phosphate buffer pH 7.4 for 1 hour before the investigation. A sufficient amount of nanoparticles, equivalent to 100 mg of SB, were put into the inner part of the dialysis membrane bag and immersed in the receptor compartment, which contained 300 ml of Phosphate buffer pH 7.4. The entire system was agitated at 100 rpm and kept at 37.50°C. 3 mL samples were removed from the receptor compartment via the side tube at defined time points (0,2,4,6,8,12,24, and 48 hours) and replaced with equal amounts of new release medium to maintain constant volume and sink conditions. After adequate dilution, the obtained samples were analysed using HPLC [37].

#### Release kinetics

The DD-Solver software application was used to investigate the mechanism of drug release from nanoparticulate formulations. For this analysis, zero order, first order, Higuchi, and Korsmeyer-Peppas were chosen. The model with the best fit was chosen along with its high correlation coefficient value [38, 39].

#### *In vitro* cytotoxicity

RPMI media supplemented with 10% (v/v) foetal bovine serum and Gentamycin Sulphate (10 g/mL) were used to grow A549 cells (NCCS, Pune, Maharashtra, India). The cells were maintained at 37°C in an incubator with a humidified atmosphere of 5% CO_2_ and 95% air. A549 cells were seeded in 96-well plates at a density of 10,000 viable cells per well and incubated for 24 hours to enable cell attachment. For 24, 48, and 72 hours, the cells were incubated with SB-loaded PCL / Pluronic F68 Nanoparticles, SB-loaded PCL nanoparticles, Paclitaxel at 0.025, 0.25, 2.5, 10, 25, and 50 mg / mL equivalent concentrations of Silibinin, and empty nanoparticles of PCL / Pluronic F68 (PCL / F68) at the same nanoparticle concentrations. Later the formulations were replaced with MTT (5 mg/mL) and cells were incubated for an additional 4 h at 37°C. Media containing MTT was aspirated and 100μL of DMSO was added for dissolved the formazan crystals [40]. A microplate reader was used to test absorbance at 570 nm (ELX 800, Biotek, USA). Using Eq 4 below, the percent cell viability was determined using untreated cells as 100%. The findings are expressed as a percentage of viable cells relative to a control group’s survival.


$$Percent\;cell\;viability=\frac{Absorbance\;test}{Absorbance\;control}\;x\;100$$ (4)

#### Intracellular ROS measurement using fluorescent DCFDA dye

Florescence microscope was used to measure the generation of intracellular ROS using the oxidative sensitive fluorescent DCFDA probe. DCFDA goes passively into the cells and interacts with cellular ROS to form a strongly florescent dichlorofluorescein compound. In brief, five different concentrations (0.025, 0.25, 2.5, 10, 25 and 50 μg / mL) of SB loaded PCL / Pluronic F68 Nanoparticles and one untreated well (control) were treated with A549 cells for 24 h. The cells were washed twice with sterile PBS after the treatment, and incubated with a culture medium containing 50μM of DCFDA for 1 h in the dark. The cells were then washed with sterile PBS to extract extracellular dye and harvested for three times. In sterile PBS the collected cells have been resuspended. The cell image was obtained using a florescent microscope (Leica DM6B, Lecia microsystems) with an excitation wavelength of 488 nm and an emission wavelength of 525 nm [40].

#### In vitro clonogenic assays

A549 cells were first seeded on 60 mm petri plates to investigate the impact of formulations on in vitro colony formation. This study included three different treatment groups: media control, blank nanoparticles, and SB loaded PCL / Pluronic F68 Nanoparticles. Following cell seeding, the cells were treated with the respective suspensions. The dishes were then left undisturbed at 37°C for 10 days. The cells were washed well with PBS, fixed, and stained with crystal violet staining at the end of the 10-day time span. A light microscope was used to count the number of colonies in each dish [41].

#### Cell cycle analysis

The Guava^®^ Cell Cycle reagent kit was used to conduct the cell cycle analysis. A549 cells were seeded at a density of 1 x 10^5^ cells per well in 6-well plates. After 24 hours, the cells were incubated again for the next 24 hours with SB solution (10 μM) and SB loaded PCL/Pluronic F68 nanoparticles. After that, the cells were fixed in 70% ethanol and stained with propidium iodide according to the protocol. Data are obtained and analyzed using flow cytometer (Millipore Corporation, USA) [42].

#### Apoptosis induction analysis

The apoptosis induction analysis was assessed by flow cytometry using Guava Nexin Kit. A549 cells were seeded in 6-well plates (1 × 10^5^ cells/well). After 24 h, the cells were then incubated for 24 h with SB solution (10 μM), SB loaded PCL/Pluronic F68 nanoparticles. Cells were washed with PBS, harvested, and centrifuged at 800 x g for 10 minutes after 24 hours of treatment. After that, 1.0 x 10^5^ treated cells were stained and analyzed in a Muse Cell Analyzer according to the manufacturer’s instructions (Merck Millipore, Darmstadt, Germany) [42].

#### Formulation of inhalation powder and characterization

The geometrical dilution method was used to combine the optimized formulations with anhydrous inhalable grade lactose in a 1:1 ratio to increase the flow property and transform them to an inhalable type. Angle of repose, Carr’s index, and the Hausner ratio were calculated [43]. In vitro release experiments were used to further characterize the optimized inhalable formulations.

#### In vitro pulmonary deposition

An eight-stage cascade impactor was used to calculate the mass median aerodynamic diameter (MMAD) of the optimised formulations [44]. Manufacturer-calibrated effective cutoff diameters for each impactor stage are as follows: stage 1 (5.8m); stage 2 (4.7m); stage 3 (3.3m); stage 4 (2.1m); stage 5 (1.1m); stage 6 (0.7m); stage 7 (0.4m). The inhaler first flowed through the cascade impactor at a rate of 28.3 L/min. Silibinin content was calculated using HPLC after the formulation was deposited in each chamber. To calculate the MMAD and geometric standard deviation, the estimated drug content in each chamber was incorporated into the MMAD calculator (GSD) [45].

#### *In vivo* study

*Animals*. Sprague-Dawley rats weighing 200–300 gm, were housed in polypropylene cages under normal temperature conditions (25 ± 1° C), relative humidity (55 ± 10%) and normal food and water ad libitum. All experimental protocols were reviewed and approved by the Institutional Animal Ethics Committee (IAEC), constituted as per guidelines of the Committee for Purpose of Control and Supervision of Experiments on Animals Government of India (IAEC/DPS/SU/1705; dated 12th December 2016) prior to initiation of the experiment.

#### NNK lung cancer model

Lung cancer model was induced by the method described by Shilpa Bhatnagar et al. and was modified as per study requirement [46]. The rats were injected subcutaneously with a single dose of 2.0 mg/kg body weight of NNK followed by repeated reduced doses of 1.0 mg/kg body weight three times a week for a total dose of 100 mg/kg body weight after one month. The experiments were terminated after 20 weeks. During the animal study to minimize pain and distress of the animals proper anaesthesia were used. During the induction phase of cancer animals were continuously monitored regarding the activity such as weight loss, food Intake and Water Intake as well as also noticed the animal behaviour through out the study.

Four different studies such as Antitumor activity, Biodistribution study, pharmacokinetic study and colloidal stability study were performed by using cancer induced animals.

#### Assessment of anti-tumor activity

After the induction of tumor Rats were randomly divided into four groups namely Control, SB, SB—loaded PCL Nanoparticles and SB-loaded PCL / Pluronic F68 Nanoparticles with 06 animals in each. Numbers of animals were decided according to the described by Shilpa Bhatnagar et al. [46]. Control animals were injected with saline on the same schedule. Prepared SB loaded PCL Nanoparticles and SB loaded PCL/ Pluronic F68 Nano formulations at a dose equivalent to 1.8 mg/kg body weight of SB, respectively was given for two months for three days in a week by micro spray aerosolizer. Rats were routinely observed for the difference in tumor size at days 0, 3, 12, 18, 24, and 30. The size of the tumor mass was calculated using a vernier caliper. Tumor volumes were determined using the following formula:

$$\text{Tumor}\;\text{volume}\;\left({\text{mm}}^{3}\right)=¼\;\text{x}\;\text{length}\;\text{x}\;{\text{width}}^{2}$$

[47].

#### Dose calculation

Human lung volume 4.5 lit was used to determine the pulmonary dose of Human SB [48]. For maintain plasma concentration above the minimum inhibitory concentration of 48 h targeted concentration for SB nanoparticles was selected as 2μg /mL. From the human dose equivalent dose calculation for rat. Targeted concentration for SB nanoparticles formulations selected as 2μg / mL to maintain plasma concentration above minimum inhibitory concentration (MIC) of 48h. The equivalent dose for rats was calculated using the human dose as a starting point. Dose calculation for rats of same surface and weight, with no deleterious effects reported at 7.7 mg/kg body weight [49]. Three different studies such as Biodistribution studies, colloidal stability study and pharmacokinetic study were performed by using animals. Silibinin-Sol (2.0 mg/kg) was injected through the tail veins and Optimized formulation were given through the Modified powder inhaler delivery device of the rats respectively [49]. By pulling the tongue outside, formulations were sprayed in the trachea region. For each phase, three animals were chosen to obtain statistically relevant data.

#### Biodistribution of SB in tumors

For tissue distribution study, organs to be analysed (liver, spleen, lung, and kidney) were removed from the study rats after they were anaesthetized intraperitonially with ketamine (80 mg/kg). To collect samples, rats were sacrificed at 2, 3, 4, 6, 8, 12, 24, and 48 hours after injection. Blood samples were withdrawal from the ocular artery and placed in test tubes containing a 10 μL of heparin solution. Plasma was separated immediately by centrifugation and held at—20°C until used. Organs from rats in each treatment group were pooled, weighed accurately, and homogenised (Ika high speed homogenizer). Plasma and tissue homogenates (0.5 mL) were mixed with 0.1 mL methyl testosterone methanol solution (8.66g/ mL) as an internal standard to determine SB. The mixture was vortexed for 5 minutes with 3 mL acetoacetate before centrifuging for 10 minutes at 3000 rpm. Two millilitres of supernatant were transferred to a test tube and dried at 45°C with airflow. The dried residue was dissolved in 0.2 mL methanol before being centrifuged for 10 minutes at 12000 rpm. Concentration of SB in each organ at a specific time was determined from the supernatant using Validated HPLC method [50].

#### Colloidal stability

Plasma, serum, liver, kidney, brain, spleen, and lung homogenates were tested for colloidal stability. Anaesthetized animals’ blood was collected from the retro orbital sinus, and plasma and serum were separated by centrifugation at 3000 rpm for 10 minutes. Tissues were homogenised in 2 mL of phosphate buffer pH 7.4 and centrifuged for 10 minutes at 4°C at 10000 rpm [50]. The supernatant was collected and diluted 1:10 (v/v) with deionised water and used for colloidal stability study. The particle size of SB Loaded PCL/Pluronic F68 NP was estimated at 2,4,6,8,12,24,48 hours after it was applied to the biological solutions.

#### Pharmacokinetic study

Sprague-Dawley (200–300 gm) of either sex, were selected for the pharmacokinetic study. The animals for the study were kept under controlled environmental conditions of 25 ±1°C temperature, 50–55% relative humidity, and 12–12 h light dark cycle in cages. The rats had free access to feed and water *ad libitum*. Animals were divided in three groups. Group I, II and III were treated with pure SB, prepared SB loaded PCL/ Pluronic F68 Nano formulations by IV and prepared SB loaded PCL/ Pluronic F68 Nano formulations by Micro spray aerosolizer at a dose equivalent to 1.8 mg/kg body weight of SB, respectively. Blood was taken at a predetermined time interval from retroorbital plexus at 0 (predose), 0.5, 1, 2, 3, 4, 6, 8, 12, 24 and 48 h. Blood was collected in iced cool heparinized eppendorf tubes. Plasma sample was obtained by centrifuging the samples (Centrifuge 5418R, Eppendorf AG, Germany) at 10,000 rpm for 20 minutes at 4 °C. Drugs from rat plasma quantified using HPLC analysis (Shimadzu Corporation, Japan) [51].

#### Data analysis

All pharmacokinetic parameters of prepared formulations were expressed as mean ± Standard Deviation (SD). The collected experimental results were analysed using ANOVA, with a p-value of less than 0.05 considered significant. The data were analysed by Graph Pad Prism (8.0.0) for further analysis.

#### Stability study

The optimized batch of Inhalable SB Loaded PCL/Pluronic F 68 NPs was used to conduct accelerated stability studies in accordance with the International Conference on Harmonization (ICH) Q1A(R2) guidelines and a method previously described by Chalikwar et al. [52]. Long-term stability and accelerated stability studies for dosage forms to be stored in a refrigerator should be performed at 5 ± 2°C and 25 ± 2°C /60 ± 5% RH, respectively, according to ICH guidelines. The primary goal of the accelerated stability analysis was to evaluate the stability of formulations at 25 ± 2°C/60% RH for 48 hours in terms of particle size, % EE, and % CDR. The freeze-dried Inhalable SB Loaded PCL/Pluronic F 68 NPs powder was poured into amber-colored glass vials and stored for 6 months at 25 2°C/60 5% RH in a stability chamber (Remi Instruments Ltd., Mumbai, India). In the same way, samples for long-term stability investigations were kept in the refrigerator at 5 ± 2°C. With a three-month sampling interval, the sample was redispersed in deionized water and tested for particle size, percent EE, and percent CDR at 48 h [53–55].

## Results and discussion

An aspect of the Statistical design was introduced to provide the Quality attribute with comprehensive Independent parameters based on the few preliminary literature studies and reviews. The optimization of these parameters could lead to the robust formulation of nanoparticles and to hit the target and to reduce the variability and to minimize or maximize the responses that increase the % Entrapment Efficiency or decreases particle size.

### Effect on particle size

**Fig 1:**
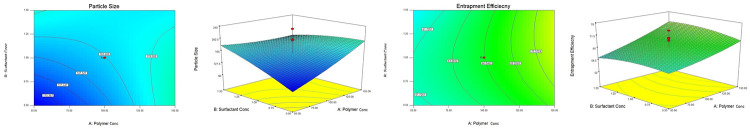
Contour and response surface plot for particle size and entrapment efficiency.

To determine the physical stability of the formulation particle size and its distribution play an important role. Obtained response was shown in Table 1. The mean particle size of the prepared Nanoparticles were found to be in between the range 108±3.21–397±3.19 nm. Particle size was increased with increasing the polymer concentration, as polymer leads to an increased the viscosity of polymer solution. Dispersion of organic phase into the aqueous phase was slow down due to the high viscosity of polymer solution, may lead to the formation of larger particles [56, 57]. As the concentration of surfactant increased, the particle size decreased, causing an increase in shear stress and viscosity, preventing the internal phase from flowing into the continuous phase. Higher polymer concentrations increase the chances of particle agglomeration, which reduces emulsifying efficacy. Nonionic emulsifiers, especially PVA, provided additional steric stabilisation, preventing small particles from accumulating in the colloidal system [58]. PVA can serve as a co-emulsifier in the manufacturing process, resulting in smaller particle sizes and better distribution. There would be differences between particles at higher surfactant concentrations, which prevents particle agglomeration and reduces particle size. An increase in Ultrasonication time, decrease in particle size because ultrasonic waves prevent the aggregation of particles as well as it converts the coarse droplet into the small droplet. The effects of factors on particle size were presented in the form of response surface plots as shown in Fig 1.

F value of quadratic model is 14.28, implies that model was significant (Table 2). Model term was significant was indicated by P value (0.0014). The R^2^ value of the regression coefficient was 0.9952, and the corrected R^2^ value was 0.9841, indicating that the experimental model had minimal variances.

For given levels of each factor, the polynomial equation in terms of coded factors was utilised to make particle size predictions.


$$\text{Y}1\;=72.83\;+\;59.18{\;\text{X}}_{1}–\;32.83{\;\text{X}}_{2}–\;22.814{\;\text{X}}_{3}–\;12.99{\;\text{X}}_{12}-3.89{\;\text{X}}_{13}–\;67.39{\;\text{X}}_{23}+\;68.21{\;\text{X}}_{1}{}^{2}–\;13.23{\;\text{X}}_{2}{}^{2}–\;5.47{\;\text{X}}_{3}{}^{2}$$ (5)

As seen in Equation when the coefficient values of the independent variables (X1, X2 and X3) were compared without the sign, the coefficient values for variables were 59.18, 32.83 and 22.81 respectively. This means that the amount of polymer had a larger influence on particle size than the amount of surfactant or the sonication time. In terms of the sign of the coefficient of variable, the amount of polymer (X1) had a positive impact on Y1, while the amount of surfactant (X2) and the sonication time (X3) had a negative impact on Y1. The reduction in surface tension and facilitation of partitioning during homogenization by increasing the concentration of PVA, which may lead to decrease in particle size [59]. Particle size reduction is accompanied by a significant increase in surface area. As a result, the primary coverage of nanoparticles’ newer surfaces competes with particle agglomeration on the exposed surface. Hence, augmentation of the PVA concentration in the primary dispersion resulted in rapid coverage of the newly formed particle surface [60]. From the p value linear coefficient terms X_1_, X_2_, and X_3_ and interaction coefficient X_23_ and X_13_ was significant.

### HPLC method development

According to ICH guideline Q2 (R1), a simple HPLC method was developed and validated for estimating SB in the prepared sample. The retention time (Rt) for the SB standard solution was 7.8 minutes according to the HPLC chromatogram (S1 Fig in S1 File). S1 Table in S1 File lists the HPLC conditions that were used (see in S1 File). Their applicability in the quantification of SB in the prepared formulation was adopted by the developed method.

### Effect on entrapment efficiency

% Entrapment efficiency was found in between the range of 52.51±1.31–81.08±1.28% (Table 1). It was observed that % Entrapment Efficiency was increased with increasing the polymer concentration. This effect of polymer concentration upon entrapment depends on drug miscibility in the organic solution and drug-polymer interaction.

This may be due to a rise in polymer concentration; the polymer in a highly concentrated solution precipitates quickly on the surface of the dispersed phase, preventing drug diffusion across the phase boundary; and the concentrated polymer solution forms a highly viscous solution, causing drug diffusion through the polymer droplets to be delayed. It could be owing to the highly positioned polymer coating, which limits its entrapment because the precipitation time was longer. As the concentration of surfactant is increased, the % Entrapment efficiency was also improves. This may be due to the presence of free drug on the nanoparticle’s surface rather than entrapment. When the ultrasonication time was increased, the high energy produced by ultrasonication helped to prevent drug from leaching out of the particles, resulting in a higher percent entrapment efficiency [61, 62]. The same relation was revealed by contour and response surface plot as shown in Fig 1.

F value of quadratic model is 12.24, implies that model was significant (Table 2). Model term was significant was indicated by P value (0.0021). The regression coefficient value R^2^ value was 0.9975 and adjusted R^2^ value was 0.9854 indicated that there was minimum variations in the experimental model.

The polynomial equation in terms of coded factors was used to make predictions about % Entrapment Efficiency for given levels of each factor.


$$\text{Y}=89.32+12.14{\;\text{X}}_{1}-\;20.24{\;\text{X}}_{2}+\;15.210{\;\text{X}}_{3}–\;2.145{\;\text{X}}_{12}–\;4.21{\;\text{X}}_{13}+\;56.32{\;\text{X}}_{23}+\;81.25{\;\text{X}}_{1}{}^{2}+\;11.32{\;\text{X}}_{2}{}^{2}+\;6.54{\;\text{X}}_{3}{}^{2}$$ (6)

When the coefficient values of the independent variables (X1, X2, and X3) were compared without the sign, the coefficient values were 12.14, 20.24, and 15.21, respectively, as shown in Equation. This indicates that the amount of surfactant had a greater influence on % EE as compared with the amount of polymer and sonication time. Considering the sign associated with the coefficient of variable, the amounts of polymer (X_1_) and sonication time (X_3_) had a positive and amount of surfactant (X_2_) had a negative impact on Y_2_, respectively.

This might be explained by the partition phenomenon, in which higher surfactant levels increased the partitioning of SB from the internal phase to the external phase medium [60]. From the p value linear coefficient terms X_1_,X_2_, and X_3_ and interaction coefficient X_12_ and X_23_ was significant.

### Optimization and validation of model

**Fig 2:**
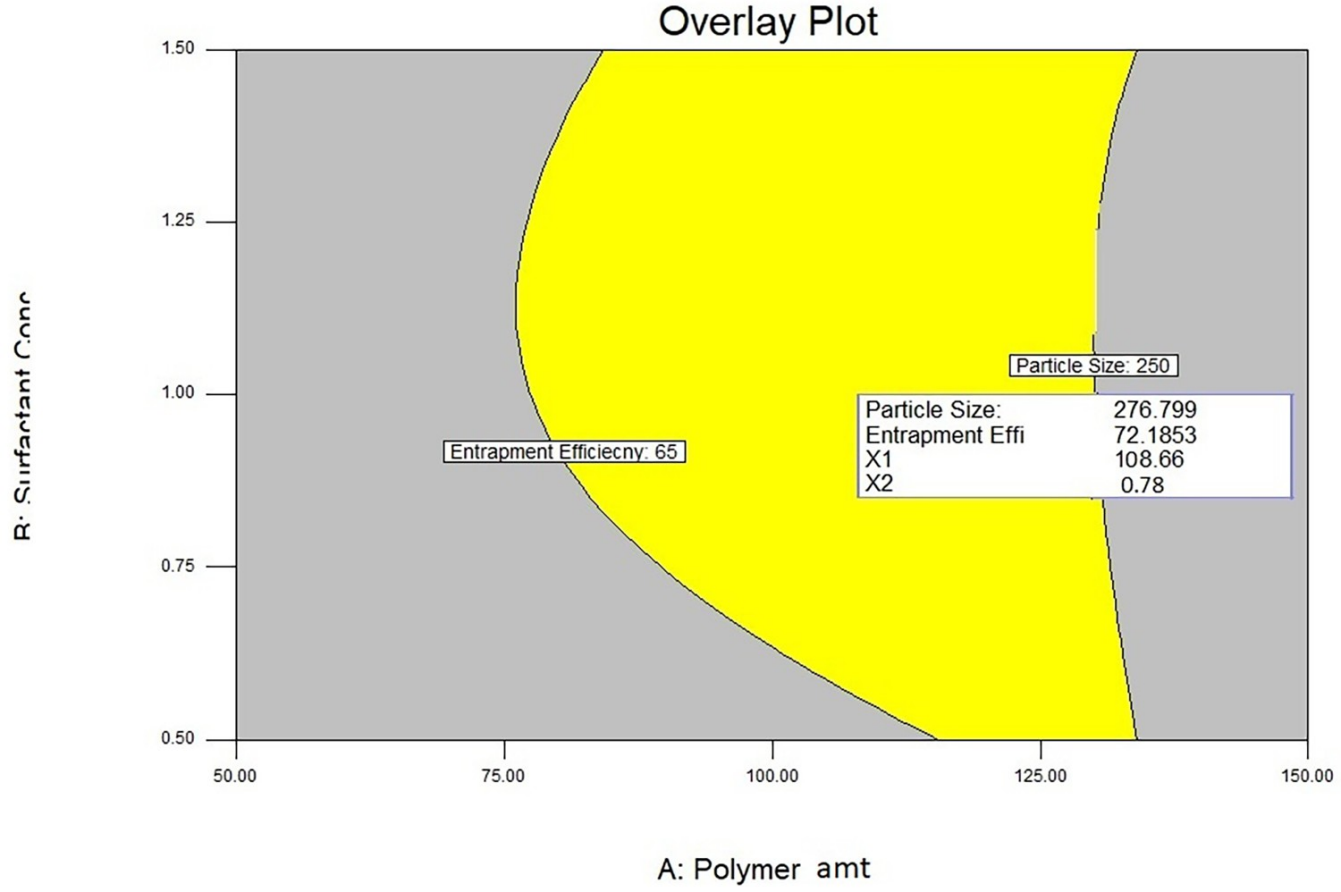
Over lay plot for check point batches.

The dependent variables (responses) were Y_1_ Particle size & Y_2_% Entrapment efficiency were found to be significant. Design expert software was used to build polynomial equations in terms of coded variables. Formulation and characterization of nanoparticles at the checkpoint batch suggested by the programme were used to validate DoE trials for formulation variables. Fig 2 shows the overlay plot suggested by DoE software displaying the design space and optimized parameters as checkpoint to obtain the desired responses. The observed values (particle size 273.45 nm and EE 71.21.68%) were comparable to the predicted values (particle size 276.23 nm and EE 72.182.12%), indicating the optimization procedure’s reliability. Also in both responses it shows an error of less than 8%. (See Table 3).

**Table 3:** Composition of check point batch.

Batch no.	X_1_	X_2_	X_3_	Response	Actual value	Predicted value	% Error
CP_1_	108 mg	0.78%	7.7min	Y_1_	273±2.45 nm	276 ±1.23 nm	1.34
				Y_2_	71.2±1.68%	72.18±2.12%	1.56

### Desirability

The Design Expert 11.0.0.0 Software solutions were sorted in order of desirability values, and the formulation with the highest desirability was chosen for optimization. For each response, the desirability value ranges from 0 to 1. One represents the ideal scenario, while zero means that one or more responses are outside the acceptable range. Desirability of the nanoparticle’s optimized formulation is 0.954. As a result, there are no chances of making an error.

### Characterization of PCL/Pluronic F68 Nanoparticles

#### Polydispersibility Index (PDI)

Size distribution of the formulation is shown by the PDI. In this study, PDI was found to be in the range 0.0201±0.10–0.254±0.21. Its indicate formulation shows uniform particle size distribution [63].

#### Zeta potential

The electrostatic repulsion between the particles will significantly influence the stability of the particles in suspension due to the zeta potential, or surface charge. The zeta potential is a measurement of nanoparticle stability. The greater the zeta potential, the more stable the formulation. For moderate stability, the zeta potential should be in the range of -30mV to +30mV. It’s also vital to determine how they interact with the cell membrane in vivo, which is generally negatively charged. Furthermore, we can estimate the dominated portion on the particle surface using the zeta potential measurement. Zeta potential of all batches ranging in between -11.74±1.54 to -15.86±1.47 mV indicate more stability of the formulation. which confirms that the system remained stable without aggregation [64]. PVA is nonionic, the increase in surface charge indicated the existence of a PVA layer on the surface, which moved the diffusive layer’s shear plane to a longer distance.

The negative surface charge of the nanoparticles formed can be attributed to the carbonyl group of the PCL polymer present at the nanoparticle structure’s surface. However, to ensure particle stability and avoid aggregation, a high absolute value of zeta potential is needed [65].

#### *In vitro* drug release study

**Fig 3:**
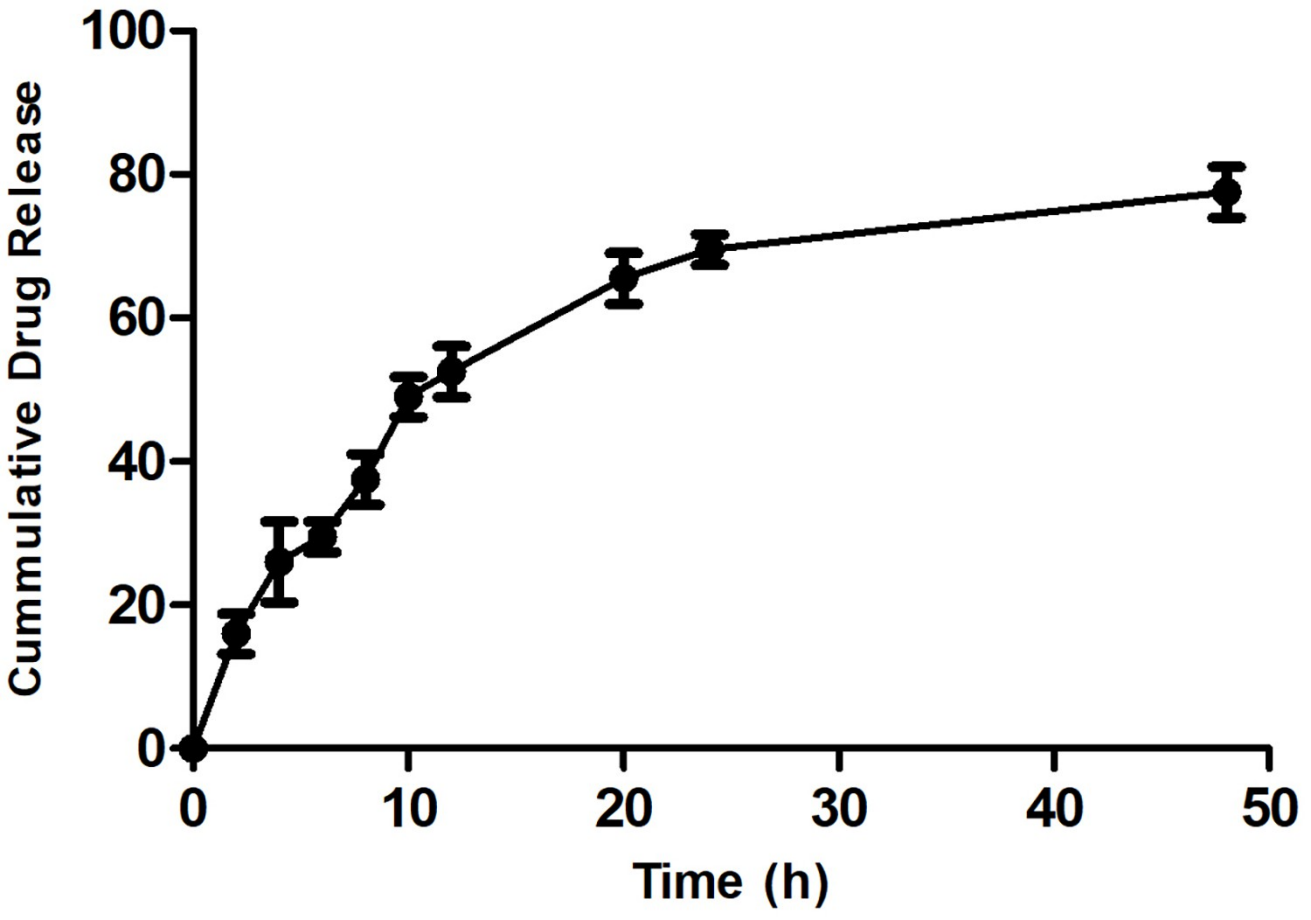
*In vitro* drug release of optimized batch. (n = 3).

In-vitro drug release was carried out by the dialysis bag diffusion techniques. The drug release study of Nanoparticles indicate that the release of drug is influenced by the concentration of polymer and size of nanoparticles. In comparison to the comparatively larger size of the particle from a higher concentrated solution, where drug diffusion is hindered due to the lower surface area of the larger particle, the quick release of drug from a low concentration of polymer was due to lower particle dimension resulting from higher surface area and thus facilitating drug diffusion. As a result, the size of the drug embedded in nanoparticles can be managed to regulate drug release. The results of the in vitro release studies of SB from Optimized NPs formulations in Phosphate buffer solution, pH 7.4, are shown in Fig 3. In the first two hours, there was an initial burst of 12–15%. The cumulative drug release from PCL/Pluronic F68 nanoparticles after 48 hours was found to be nearly 80%. In contrast to the control, all nanoformulations showed a sustained release effect. The drug molecules inside the matrix have a shorter average diffusion path due to the nanosize particles, which allows faster diffusion.

Following that, the release rate decreased, indicating not only the regulation of drug release by drug diffusion through the polymer matrix, but also the problem of drug degradation after 24 hours of loading. As a result, it was clear that incorporating Silibinin into PCL/Pluronic F68 nanoparticles might significantly sustain the Silibinin release [66–68].

To evaluate mechanisms for drug release, the results of the in vitro release were applied to different kinetic models using the software programme DD-Solver Excel Sheet. Korsmeyer-Peppas order kinetics was found to be the best fit model for the optimized batch. The correlation coefficient for the Korsmeyer-Peppas model was found to be high when the graph was plotted between log time and log percentage drug remaining. Since the release exponent value (n) is 0.401, the Korsmeyer-Peppas model is the best fit for nanoparticles. The release exponent (n) was less than 0.5, indicating that SB was released from nanoparticles through non-Fickian diffusion [69].

#### Fourier Transform Infrared spectroscopy (FTIR)

**Fig 4:**
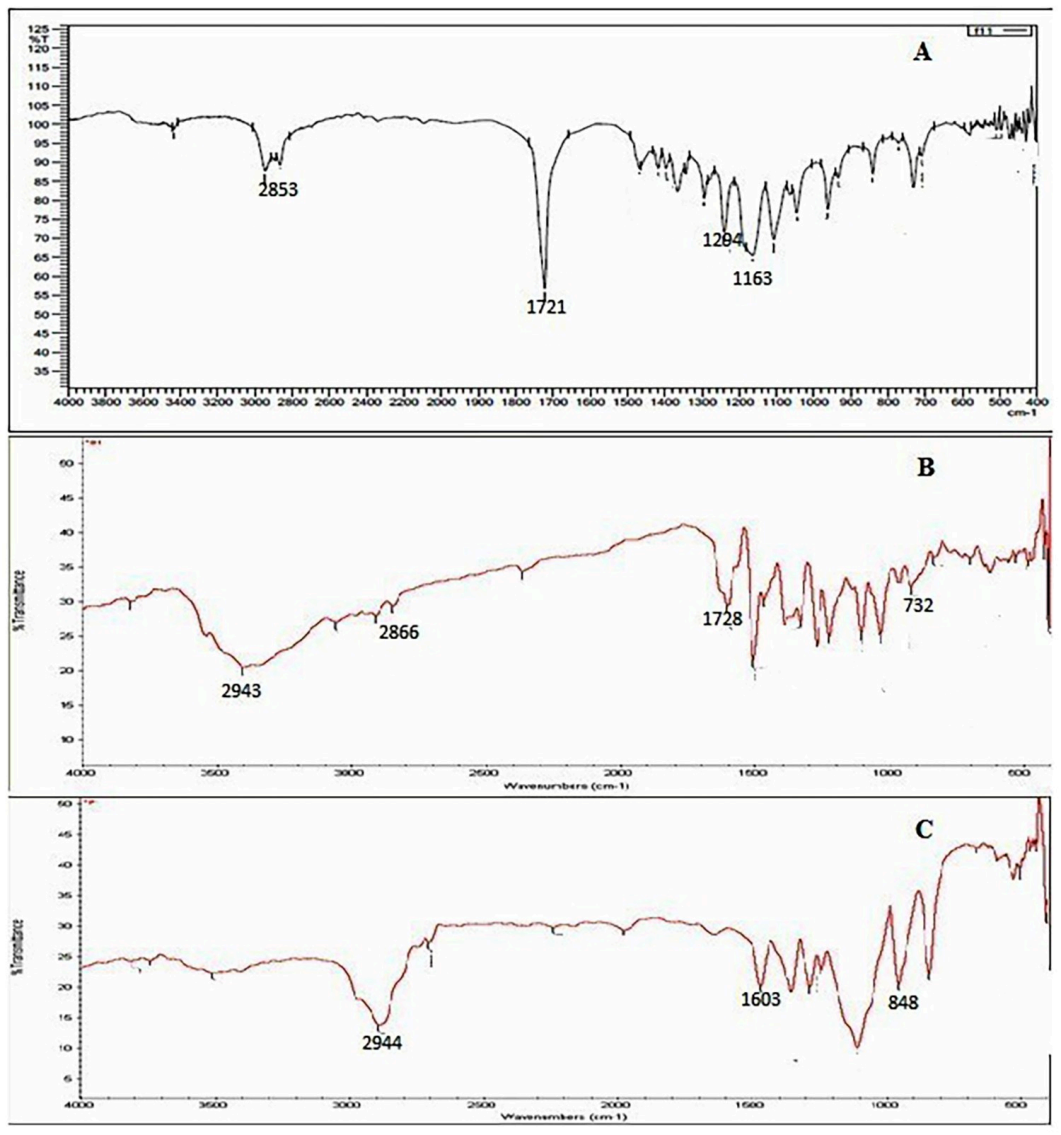
FTIR Spectrum of A) Silibinin B) PCL/Pluronic F68 and C) Prepared Nanoparticles.

FT-IR spectrum of Silibinin shows characteristic peaks at 2848.1 cm-1 (OCH3 group), 1603.3 cm-1 (C = N+ ammonium group) and 1506.6 cm-1 (aromatic C = C bending). FT-IR spectrum of PCL/Pluronic F68 showed characteristic peaks at 732.95 cm-1, 1728.22 cm-1, 2866.22 cm-1, 2943.37 cm-1 corresponding to C-O stretching, C = O stretching, Symmetric CH_2_ and asymmetric CH_2_ [67]. Its confirm the polymerization of PCL/Pluronic F 68. FT-IR spectra of Silibinin, PCL/Pluronic F68 and Silibinin loaded PCL/Pluronic F68 nanoparticle formulations were shown in Fig 4. All these functional peaks were present in the Silibinin loaded PCL/Pluronic F68 nanoparticle formulation. Thus, it confirmed that there was no significant interaction was found between drug and excipients in the formulation.

#### Differential Scanning Calorimetery (DSC)

**Fig 5:**
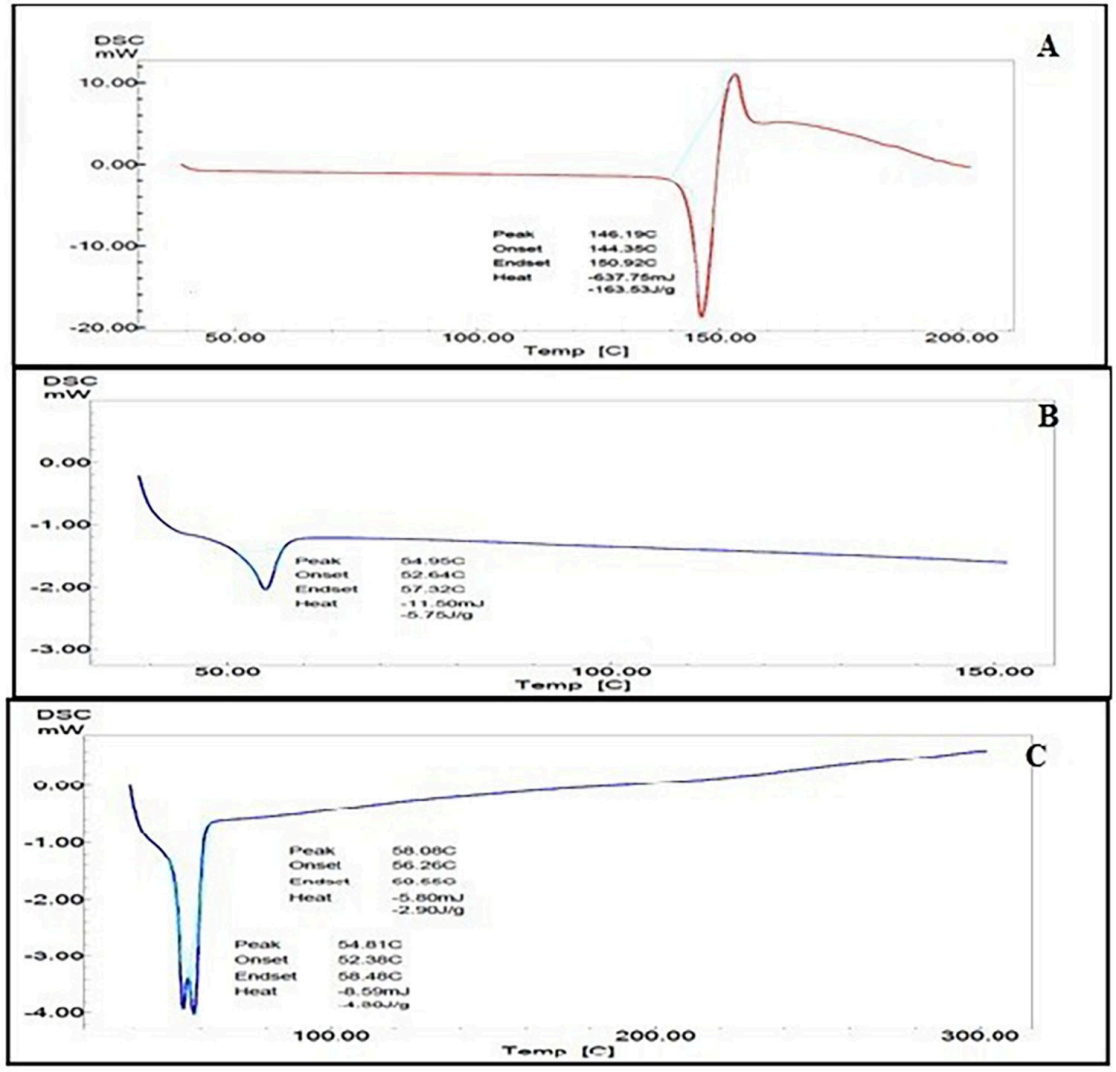
DSC Thermogram of A) Silibinin B) PCL/Pluronic F68 and C) Prepared Nanoparticles.

The DSC thermogram of Silibinin shows broad endothermic peak at 149.96°C, corresponding to its melting point. DSC thermogram of PCL/Pluronic F68 shows sharp endothermic peak at 54.95°C. DSC thermogram of nanoparticulate formulation shows complete disappearance of characteristic peak of Silibinin; a fact that the drug was molecularly dispersed within the polymeric matrix. The DSC curve of Silibinin, PCL/Pluronic F68 and Silibinin loaded PCL/Pluronic F68 nanoparticles were shown in Fig 5.

#### Scanning Electron Microscopy (SEM)

**Fig 6:**
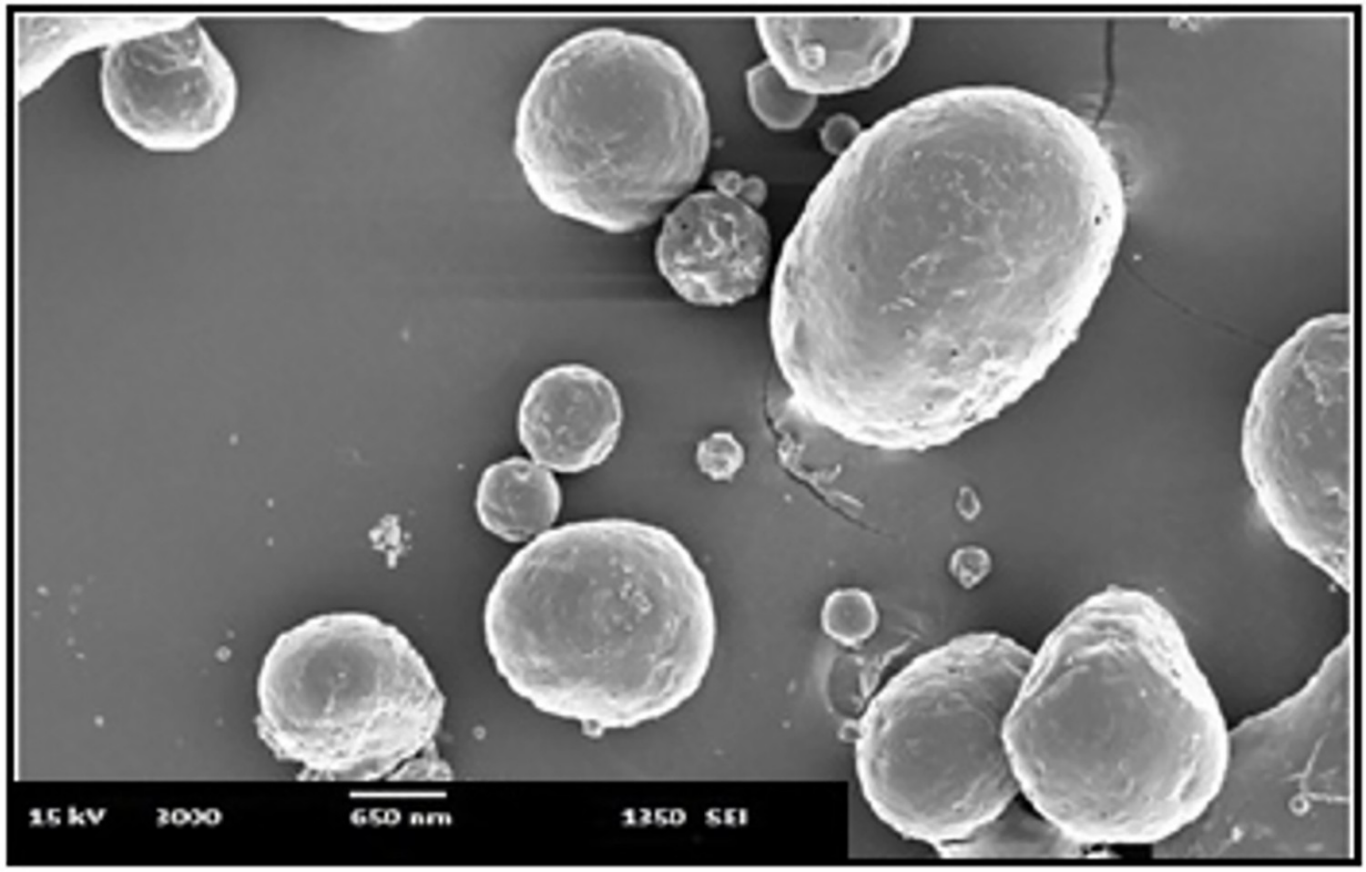
Surface morphology of optimized nanoformulation.

The morphology of the optimized batch was examined by Scanning Electron Microscopy. SEM photographs of the SB loaded PCL/Pluronic F68 nanoparticle shown in Fig 6. Nanoparticles can be seen as being spherical in shape. This nanometric size also suggests that SB Loaded PCL/ PLuronic F 68 Nanoaprticles can preferably for Inhalation drug delivery system as well as thereby enhancing the bioavailability of the SB.

#### *In vitro* cytotoxicity

The impact of loading SB in PCL / Pluronic F68 nanoparticles cytotoxicity was assessed by determining the viability of the Lung cancer A549 cells after 48 h.

**Fig 7:**
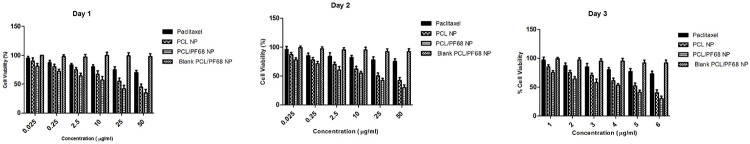
Viability of A 549 cells cultured with Silibinin loaded PCL nanoparticles and PCL/Pluronic F68 (PCL/F68) nanoparticles in comparison with that of Paclitaxel at the same and empty PCL/Pluronic F68 (PCL/F68) nanoparticles with the same amount of nanoparticles (n = 6).

Fig 7 shows the *in vitro* viability of A549 cells cultivated with Paclitaxel, PCL / Pluronic F68 nanoparticles and PCL nanoparticles at 0.025, 0.25, 0.5,1, 2.5, 10, 25 and 50 μg / mL concentrations. From this figure it can be shown that in general the drug formulated in PCL nanoparticles showed better effects on the cancer cells compared to standard paclitaxel. SB Loaded PCL / Pluronic F68 nanoparticles achieved even superior therapeutic effects than PCL nanoparticles and standard paclitaxel. Compared with Standard Paclitaxel, the higher cytotoxicity was seen in the two formulations of nanoparticles can be due to the higher cellular absorption as well as the sustained manner of drug release. Pluronics could cause a drastic sensitization of MDR tumors hence shows the higher cellular uptake and faster drug release of PCL/Pluronic F68 nanoparticles over PCL nanoparticles [70, 71]. Moreover, we gone through the mechanism of Pluronic effect in MDR cells and it has been shown that pluronic polymer can alter their microviscosity by insert into membrane, Induce a reduction of ATP level in cancer cells, Inhibit P-glyco protein drug efflux transporters with multidrug resistance [22, 71, 72]. Moreover it’s also increase the rates of reactive oxygen species in cytoplasm, decrease anti apoptotic response in MDR cells [73, 74]. Moreover, recent studies have shown that Pluronic F68 is both a strong P-gp and CYP3A4 in vitro inhibitor [75]. Also multidrug resistance of cancer cells proved with other carriers such as PEG-PCL copolymer and n-(2-hydroxypropyl) methacrylamide (HPMA) copolymer [24, 76]. IC50 which is defined as the drug concentration at which 50% of the cells in culture were killed over a specified period of time. It could be used to quantitatively evaluate the in vitro therapeutic effects of a nanoparticle formulations. Results are witnesses to proved that PCL/Pluronic F68 nanoparticles shows better cytotoxicity activity as compared to PCL nanoparticles. Table 4 shows the IC50 Value of Paclitaxel, SB Loaded PCL nanoparticles, SB Loaded PCL/Pluronic F68 nanoparticles for 24,48 and 72 h. Such advantages of the nanoparticles formulations would become even more relevant in achieving higher cytototoxicity if more attention is given to the controlled way the drug is released from the nanoparticles.

**Table 4:** IC50 of A549 cells after 24h, 48h, and 72h incubation with Silibinin formulated in Paclitaxel, PCL, and PCL/Pluronic F68 nanoparticles at various drug concentration. (n = 6).

Incubation Time(h)	IC50 (μg/mL)		
	SB Loaded PCL/Pluronic F68 NPs	SB Loaded PCL NPs	Paclitaxel
24	2.5	4.2	6.8
48	1.8	3.8	5.2
72	1.6	3.5	5

#### Intracellular ROS measurement using fluorescent DCFDA dye

**Fig 8:**
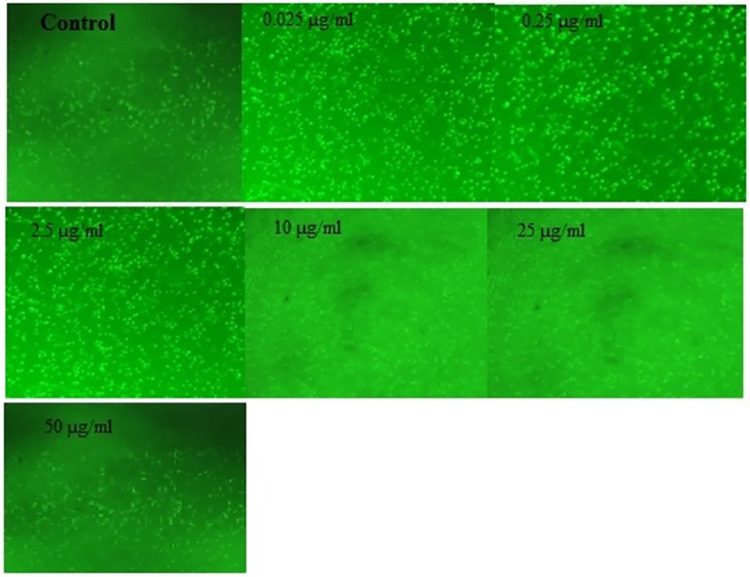
DCFDA staining images of A549 cells to measure intracellular reactive oxygen species.

**Fig 9:**
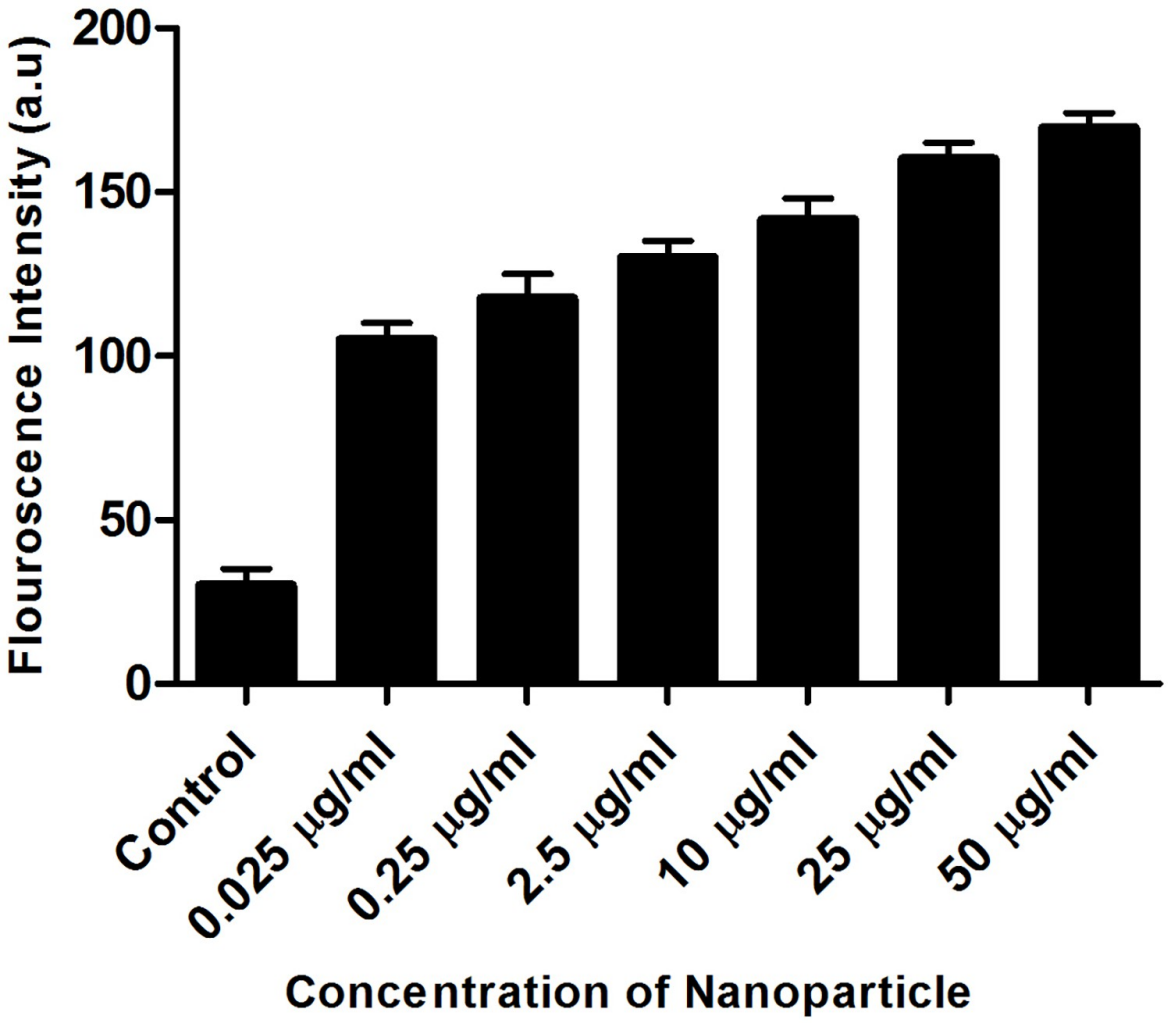
Florescence intensity graph of DCFDA staining of control cells and treated cells.

Since ROS generation is associated with apoptosis, the intercellular ROS levels in SB loaded PCL/Pluronic F68 nanoparticles treated A549 cells were investigated using oxidation-sensitive DCF fluorescence intensity observed under a florescence microscope (Fig 8) and quantified (Fig 9). The non-fluorescent DCFDA permeabilized easily through the cell membrane and, in the presence of ROS, was oxidized into highly fluorescent DCF. The increase in fluorescence intensity indicates increased formation of intracellular ROS in the cell. The PCL/Pluronic F68 nanoparticles concentration-dependent significant increase in DCF fluorescence was detected in treated cells. This shows that SB loaded PCL/ Pluronic F68 nanoparticles induces formation of ROS as an essential mechanism for apoptosis induction.

#### *In vitro* clonogenic studies

**Fig 10:**
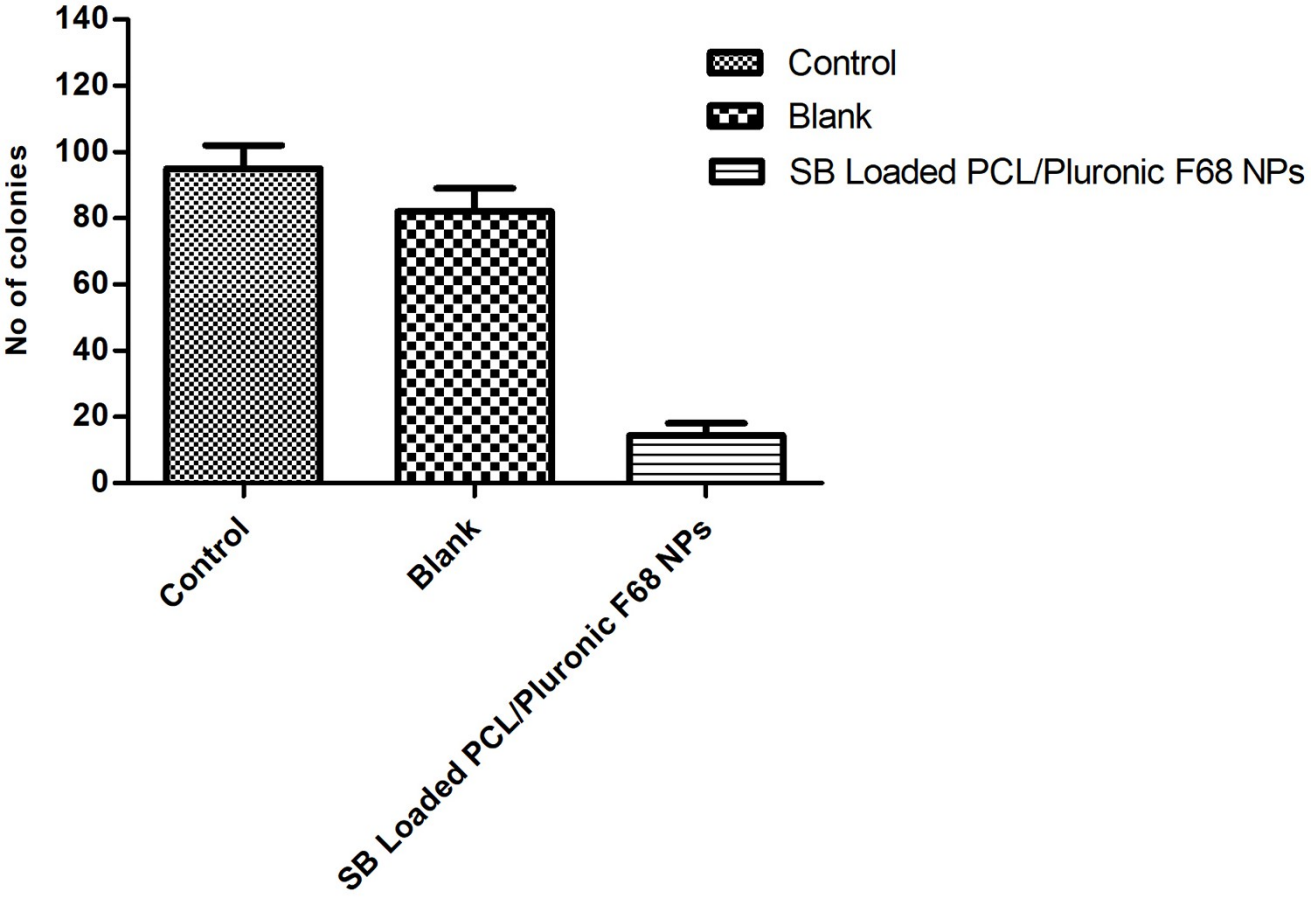
Colony forming study A549 cells.

Clonogenic test was employed to determine cell viability, which avoided the use of fluorescent or colorimetric indicators (e.g. MTT, neutral red) that could be affected by nanomaterial interference. In this experiment, cells were planted at clonal density, and the number of colonies that survived was used to calculate cell viability in response to a specific treatment. The relative change in proliferation following exposure to a given treatment was demonstrated by changes in colony size.((Fig 10) 17. Following a 5-day exposure a significant reduction in the colony number was observed, which indicated inhibition of the colony forming ability of formulations on A549 cells. SB Loaded PCL/Pluronic F68 nanoparticles showed significant reduction in colony formation to 17% compared to that of the controls. After a 5-day exposure to SB Loaded PCL/Pluronic F68 nanoparticles, a substantial reduction in colony number was observed.

#### Cell cycle analysis

**Fig 11:**
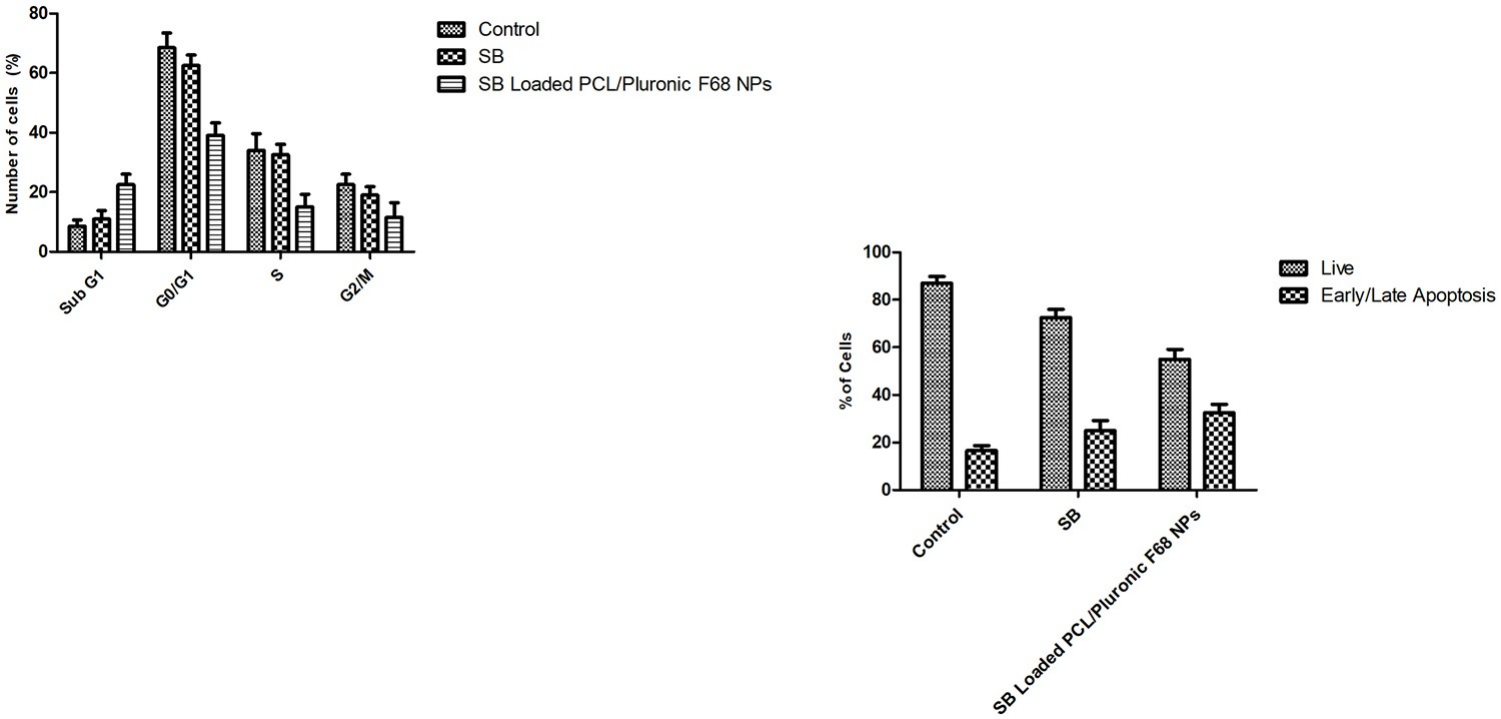
A) The number of A549 cells in each phase of the cell cycle (Sub-G1, G0/G1, S, G2/M). Cell cycle analysis was assessed flow cytometry B) Induction of apoptosis in A549 cells was examined by flow cytometry.

The percentages of A549 cells in different phases of the cell cycle were analyzed by flow cytometry after 24 h of treatment (Fig 11A). At 10 μM of SB loaded PCL/Pluronic F68 nanoparticles, 18.21% of treated cells were in the sub-G1 phase (p < 0.001), which is indicative of enhanced apoptosis. Therefore, SB loaded PCL/Pluronic F68 nanoparticles suppresses cell cycle progression due to increase of cells entering in sub-G1 phase. On the contrary, treatment with SB arrested A549 cells in the G0/G1 phase, followed by a significant decrease in the S-phase population. cytoplasmic sequestration of cyclin D1 and CDK2, contributing SB induced G1 arrest [77, 78].

#### Apoptosis induction analysis

Apoptosis-inducing effect of SB loaded PCL/Pluronic F68 nanoparticles on A549 cells was performed by an annexin V-phycoerythrin (PE) assay after 24 h of treatment (Fig 11B). In agreement with flow cytometric results, A549 cells treated with SB loaded PCL/Pluronic F68 nanoparticles showed 51.12% of apoptotic cells (early and late apoptosis) as compared with the untreated control group (p < 0.001). In contrast, cells treated with SB did not show any significant changes in apoptotic cell death (p > 0.05). SB loaded PCL/Pluronic F68 nanoparticles demonstrated 3.2-fold induction in apoptosis as compared with free SB solution [79].

#### Formulation and characterization of inhaled powders

Inhaled chemotherapy has been shown to respond well against lung cancers. Inhalation will modify drug biodistribution and induce the accumulation of a higher fraction of the lungs when compared to parenteral delivery [79]. The development of a suitable carrier system has become a necessity for deep lung delivery, but it is still difficult to use it for pulmonary delivery of NPs. Apart from the necessary protection when it comes to contact with lung tissue, the carrier must also maintain drug stability, ease of handling during filling and processing, proper lung deposition, and the required aerodynamic properties. The main benefit is better powder flowability, which aids in drug distribution from the inhaler device [80]. Prepared optimized formulations were characterized and reported fair flow property such as angle of repose, Carr’s index, and Hausner ratio.

For improving the flow property anhydrous inhalable grade lactose was used. It has been shows that flow property of optimized formulations was remarkable increased as 26.14°, 8.52%, and 1.21 for angle of repose, Carr’s index, and Hausner ratio respectively. The fine particles of this anhydrous inhalable grade lactose may be the reason for the improvement of flow property. According to an in vitro drug release analysis, inhalable grade lactose has no impact on the release characteristics (S1 Fig in S1 File). Furthermore, nanoparticles’ sustained release properties will improve the effectiveness of inhaled chemotherapy by keeping drug concentrations at tumour sites for longer periods of time [81]. Clearly, the initial dosage form’s physicochemical properties, aerosol mechanics leading to lung deposition, and biological obstacles to presenting the drug to the circulation all play a role in the probability of efficacy in treating systemic disease.

#### *In vitro* pulmonary deposition

Anderson cascade impactor equipped with Easyhaler^®^ was used to test the aerosol performance of the nanoparticle compositions in vitro. Both the deposition site and the mass of inhaled medications deposited in the respiratory system are influenced by particle size. In step 5, the maximum level of particle was deposited (S2 Fig in S1 File). Particulate MMAD is the diameter of a sphere with unit density and has the same aerodynamic behaviour as the particle being considered. For the SB loaded PCL/Pluronic F 68 nanoparticles emitted MMAD and GSD were found to be 4.235 ±0.124 μm and 1.958±1.23 respectively. The combination of a low MMAD and a low GSD indicates a narrow size distribution, which is advantageous for targeted drug delivery. Inhalation formulations that have been created can also reach the deepest part of the lungs. Aerosols for therapeutic use Particles larger than 10 μm cannot enter the lungs; instead, they are deposited in the mouth, nose, pharynx, and larynx through impaction. Particles between 0.1 and 1 μm in diameter do not settle well in the lungs, and a large proportion is expelled. Finally, the advantages of this pulmonary DDS could include quicker drug deposition in the lungs, fewer systemic side effects, and an improved drug therapy index. Furthermore, the formulation is likely to be effective in the fight against lung cancer [82, 83].

#### *In vivo* study

Human lung volume, such as 4.5 lit, was used to measure the pulmonary dose of human SB [36]. SB nanoparticles were given a target concentration of 2 μg/mL in order to keep plasma concentrations above the minimum inhibitory concentration of 48 h. The human equivalent dose was used to measure the rat dose. SB nanoparticle formulations were given a target concentration of 2 g/mL to keep plasma concentrations above the minimum inhibitory concentration (MIC) of 48 hours. The equivalent dose for rats was calculated using the human dose. The dose calculation for rats with equal surface and weight showed that 7.7 mg/kg body weight had no adverse effects [37–39]. Animals were used in four separate experiments, including In vivo anti tumor activity, Biodistribution studies, colloidal stability studies and pharmacokinetic studies. Three groups of twelve Sprague-Dawley rats (n = 12) were randomly assigned. Group I,II and III were administered Silibinin-Sol (2.0 mg/kg) which was injected through the tail veins, SB loaded PCL Nanoparticles, and SB laoded PCL/Pluronic F68 Nanoparticles and were given through the modified powder inhaler delivery device of the rats [40]. By pulling the tongue outside, formulations were sprayed into the trachea. For each time, three animals were chosen to obtain statistically relevant data.

#### *In vivo* anti-tumor activity

**Fig 12:**
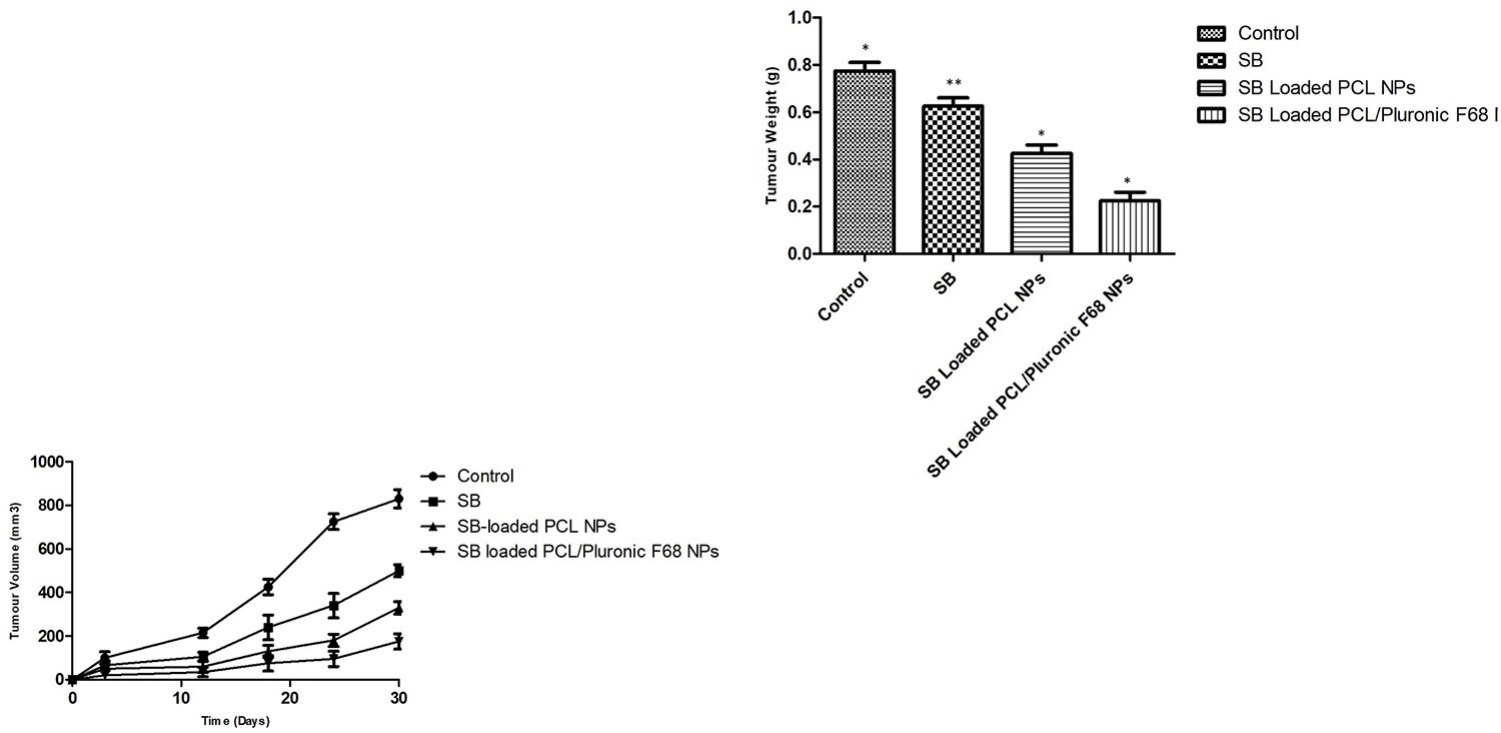
Tumor volume changes of the rat after administered of saline, SB, SB loaded PCL/ nanoparticles, and SB loaded PCL/Pluronic F68 nanoparticles. (B) Tumor weight analysis of mice after 30 days treatment. (n = 3; * * p<0.005, * p<0.05).

Standard SB, SB loaded PCL Nanoparticles, and SB laoded PCL/Pluronic F68 Nanoparticles activity against tumors were shown in Fig 12. Free medication has struggled to increase the number of tumors. It can probably be attributed to its rapid circulation clearance or less tumor-targeting capacity. SB laoded PCL/Pluronic F 68 Nanoparticles showed a greater reduction in tumor volume as compared to SB loaded PCL Nanoparticles (Fig 12A). Pluronics is a key trait, as is its ability to incorporate membranes followed by cell translocation, affecting a variety of cellular functions including mitochondrial respiration, ATP synthesis, drug efflux transporter activity, apoptotic signal transduction, and gene expression [84]. This finding may be due to SB laoded PCL/Pluronic F 68 Nanoparticles accumulation in tumors accompanied by endocytosis mediated by the receptor. This is the main reason for better anti-tumor activity in comparison with SB loaded PCL Nanoparticles. As shown in Fig 12B, tumor weight of SB laoded PCL/Pluronic F 68 Nanoparticles also reduced by 2.2 g to 0.9 gm which is evidence about the superior antitumor activity. In vivo antitumor efficacy results indicated that of SB laoded PCL/Pluronic F 68 Nanoparticles had significant potential for lung carcinoma treatment.

#### Biodistribution of SB in tumors

**Fig 13:**
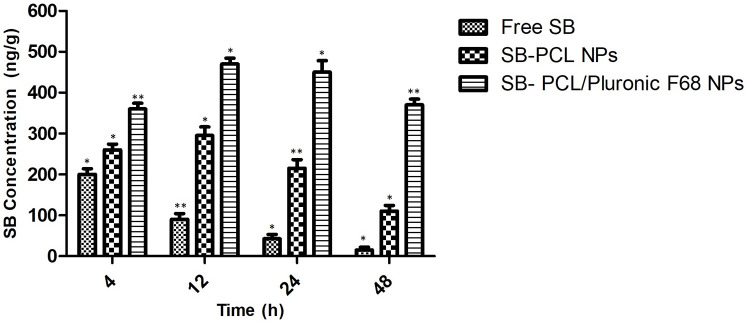
*In vivo* biodistribution of SB in free SB, PCL Loaded NPS, and PCL/Pluronic F 68 NPs groups at 4, 12, 24, and 48 h after injection, respectively. (n = 3; * * p<0.005, * p<0.05).

The tumor-targeting ability of SB Loaded PCL/Pluronic F68 nanoparticles was verified by the *In vivo* biodistribution of SB at tumor sites in A549 tumor cells bearing rats. Free SB was rapidly distributed in tumours at 3 h and rapidly eliminated at 8 h, as shown in Fig 13. In addition, after 24 h the amount of SB in the tumors site was negligible, in PCL nanoparticles and PCL/Pluronic F68 nanoparticles SB concentration exhibited a gradual increase in tumor site. In summary, the amount of SB in the PCL nanoparticles and PCL/Pluronic F68 nanoparticles groups peaked at 12 hours after injection, which was significantly higher than the free SB group. As compare to the free SB, SB Loaded PCL nanoaprticles have shown the higher amount of SB, mainly attributed to the controlled SB release from PCL nanoaprticels, which shows more SB accumulated in tumor sites and less SB released in blood circulation. It’s worth noting that at all time points, PCL/Pluronic F68 nanoparticles had a higher SB concentration than PCL nanoparticles. Maintain a high SB concentration for at least 48 hours. These findings, which are primarily due to Pluronic block copolymers, suggest that a macropinocytosis-dependent pathway may improve drug uptake in tumour sites. It’s also due to endocytosis’s rapid absorption and high permeability, which allows intracellular nanoparticles to accumulate. All of the findings suggested that coated Pluronic F 68 could effectively increase SB accumulation at tumour sites [3, 85, 86].

#### Pharmacokinetic study

Despite the fact that systemic drug delivery via nanocarriers is more successful for lung cancer than standard intravenous drug administration, direct anticancer medication delivery via inhalation has numerous additional advantages. Aerosols transmit anticancer drugs directly to cancer cells, resulting in higher therapeutic effectiveness at lower drug concentrations. The findings show a linear relationship between analyte concentration and peak area. The drug retention time was 2.84 minutes. In the concentration range of 2–28 mcg/mL, the method was found to be linear (r2 = 0.994; n = 6) [87]. One of the main objectives of the proposed research is to investigate the significant differences in pharmacokinetic impact between formulations. Table 5 shows the pharmacokinetic parameters of SB Loaded PCL/ Pluronic F 68 nanoparticles after IV and pulmonary administration.

**Table 5:** Pharmacokinetic parameters of nanoparticles in the rat after administration of silibinin, IV administration of nanoaprticles and inhalation administration of nanoparticles. (n = 6).

Parameters	Silibinin	Prepared SB Loaded PCL/Pluronic F 68 Nanoparticles by IV Route	Prepared SB Loaded Pluronic/F68 Nanoparticles by Inhalation Route
**Cmax (μg/mL)**	20.15±2.32	54.10±1.02	80.11±2.14
**T**_**max**_	4.1±2.02	4.3±1.54	6.2±2.85
**AUC**_**total**_ **(μg h /mL)**	225.54 ± 10.21	325.21±9.23	587.31 ± 14.12
**T**_**1/2**_ **(h)**	4.1±4.23	7.1±1.25	8.4±2.32
**CL (1/h)**	0.98±0.32	0.95±1.15	0.65±0.25
**MRT (h)**	5.25±1.05	5.34±1.21	6.19±2.56
**F**_**rel**_	1	1.89	4.20

**Fig 14:**
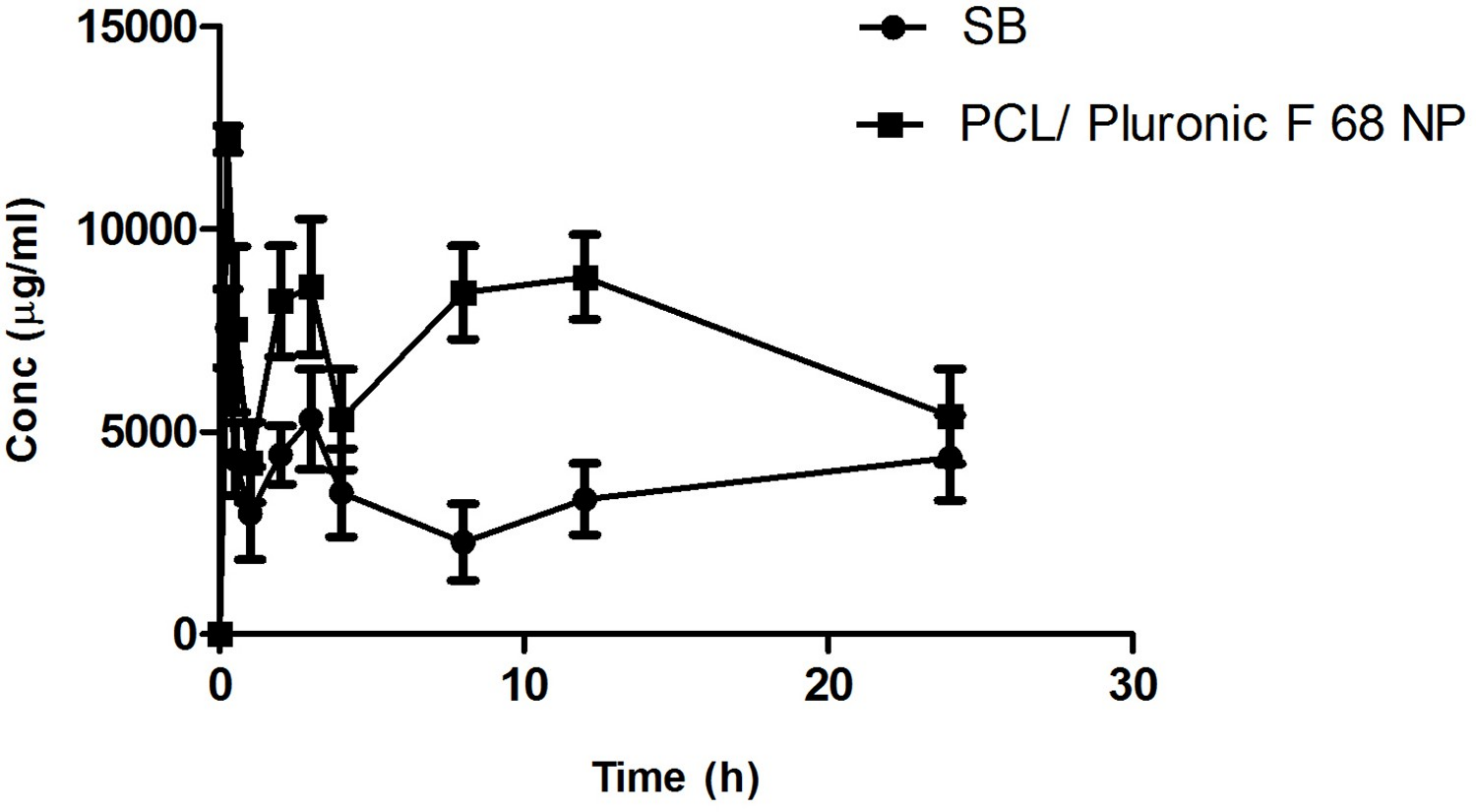
The average plasma concentration-time curves of pure drug and prepared nanoparticles (n = 6).

Fig 14 shows the average plasma concentration-time curves of prepared nanoparticles after single dose administration in Sprague Dawely rats, and Table 5 summarises the pharmacokinetic parameters. When compared to nanoparticles administered via IV route, the maximum plasma concentration (Cmax) achieved with inhalable nanoparticles was nearly four times higher. According to the above findings, Cmax of the formulation was increased as compared to IV administration, indicating an improvement in AUC, which leads to increased bioavailability. The increase in AUC was attributed to the avoiding hepatic first pass metabolism by being targeted in the intestine. Tmax was raised from 4.1±2.02 to 6.2±2.85 due to the sustained release of drug. Similarly, the Tmax of the formulation was higher than that of IV administration, indicating that the medication remains in the systemic circulation for a longer period of time, implying that sustained release was achieved. Elimination of the drug from the formulation was less compared with the formulation by IV Route. In comparison to the narrow airway and alveolar volume, the broad alveolar surface provides a strong superficial layer for pulmonary drug delivery and absorption. Passive diffusion, facilitative and active transport through solute transport carriers on the apical and basolateral surfaces of the plasma membrane, and vesicle-mediated transport all occur across the lung epithelium [88]. The pulmonary mucosal surface is naturally permeable to molecules after inhalation because it is rich in anti-protease enzymes. In contrast to intravenous injections, pulmonary delivery of nanoparticles can result in faster absorption and increased local as well as systemic bioavailability [89]. This result suggests the improved rate of absorption with the administration of prepared SB Loaded PCL/Pluronic F68 Inhalable nanoparticles. It is manifested that at each time point the plasma concentration of prepared nanoparticles was remarkably higher than those administrated with the SB solution. Relative bioavailability (Frel) was calculated as the ratio of AUC total (Inhalable nanoparticles) to AUC total (IV nanoparticles). This finding suggested that prepared Inhalable nanoparticles exhibited 4.20 folds more bioavailable than the Nanoparticles by IV Route. For instance, the administration of aerosolized doxorubicin-nanoparticles through dry powder aerosolizers has reduced cardiotoxicity, prolonged survival (up to 140 days vs. <50 days), and suppressed the lung metastasis in mice compared to the intravenous delivery of the free drug as well as doxorubicin nanoparticles [90].

#### Colloidal stability study

**Fig 15:**
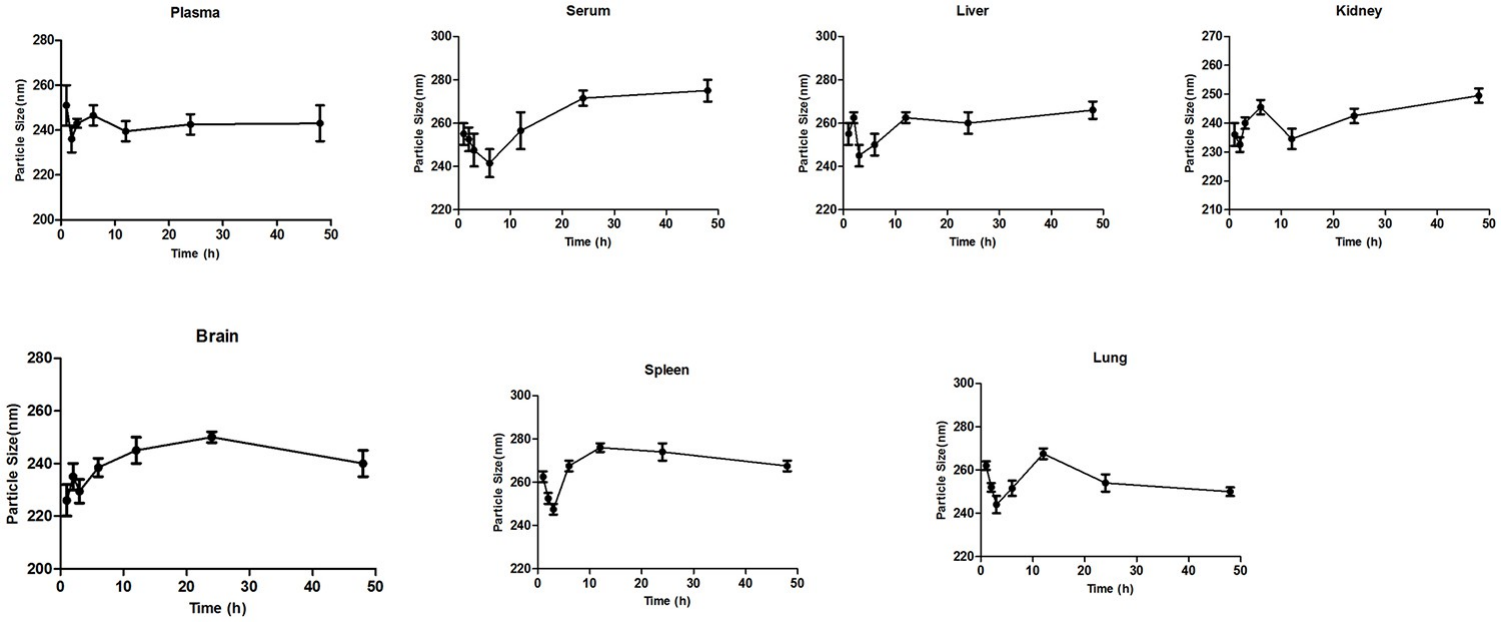
Colloidal stability study in plasma and different tissues (n = 6).

Nanoparticles possess a number of advantages when it comes to supplying chemotherapeutic agents for cancer treatment. To simulate a normal persistence in the body, NP stability in serum and tissue homogenates was studied for 48 hours. Furthermore, these nanoparticles alter the nanoparticles’ physicochemical properties. Several studies have suggested that colloidal stability is essential for successful drug delivery [91]. The interactions of NPs with the biological environment should also be investigated in order to develop stable drug delivery systems. As a result, the stability of the optimized nanoparticles was investigated in rat plasma, serum, liver, kidney, brain, spleen, and lung. The experiment was carried out at 37°C for a specific period of time. In all biological solutions, nanoparticles were found to have a consistent particle size (Fig 15). According to the findings, the nanoformulations developed might inhibit particle aggregation both at the target site and during their circulation in the body. This could be owing to the protective Pluronic F68 coating that forms during nanoparticle production. It has been found to have an impact on the physicochemical properties of nanoparticles. It can provide a steric shield on the surfaces of nanoparticles, improving the nanosystem stability [92].

#### Stability study

The aim of a stability study was to assess a product’s shelf life by accelerating the rate of decomposition. For six months, the optimized formulation was subjected to stability studies as per ICH guidelines, particle size, drug entrapment efficiecny, and drug release monitored at 48-hour intervals. The results of stability studies conducted on freeze-dried Inhalable SB Loaded PCL/ PLuronic F68 nanoparticles (S1 Table in S1 File). After 3 months at 5 ± 2°C and after 6 months and at 25 ±2°C/60 ± 5% RH there was no significant change (p> 0.05) noted in the particle size (nm), %EE and % CDR at 48 h. The storage of Inhalable SB Loaded PCL/ PLuronic F68 NPs at 25 ± 2°C/60 ± 5% RH resulted in a slight increase in particle size and a drop in % CDR after 48 h, as well as a substantial decrease in % EE at 6 months (p < 0.05). The coalescence of nanoparticles causes the increase in particle size at 25 ± 2°C/60 ± 5% RH. The increase in size and resulting decrease in surface area could explain a fall in % CDR after 24h. The percent EE of Inhalable SB Loaded PCL/ Pluronic F68 NPs decreased slightly after storage, which can be due to SB expulsion from the Nanoparticles matrix.

## Conclusion

As per our knowledge, the first attempt was done to prepared, a novel SB-loaded PCL/Pluronic F68 nanoparticle formulation was prepared for the treatment of lung cancer. In conclusion, SB loaded PCL/ Pluronic F68 nanoparticles were successfully prepared with good entrapment, smaller particle size along uniform spherical shape and good aerosolization behaviour. The optimized SB loaded PCL/Pluronic F68 nanoparticles demonstrated sustained release and followed non-Fickian diffusion-based release kinetics. Many in-vitro and in-vivo studies, in which SB loaded PCL/ Pluronic F68 nanoparticles were compared to plain drug to determine therapeutic efficacy, supported the efficacy of the proposed formulation in the treatment of lung cancer. With a better pharmacokinetic profile, developed nanoparticles may have a longer residence period and accumulate in the lungs. This study can provide a reliable platform for development of SB-loaded Inhalation nanoparticles for further preclinical and clinical studies. The outcomes of this research confined, SB loaded PCL/ Pluronic F68 nanoparticles could be an effective approach in Lung cancer targeting using inhalation route.

## Supporting information

S1 File (DOCX)

S1 Graphical abstract (JPG)
