# Supplementary material for: Lung cancer targeting efficiency of Silibinin loaded Poly Caprolactone /Pluronic F68 Inhalable nanoparticles: In vitro and In vivo study
Source: PLoS One. 2022 May 13;17(5):e0267257. doi: 10.1371/journal.pone.0267257 (PMC9106168; doi:10.1371/journal.pone.0267257)
Supplement: S1 File — (DOCX) [file pone.0267257.s001.docx]

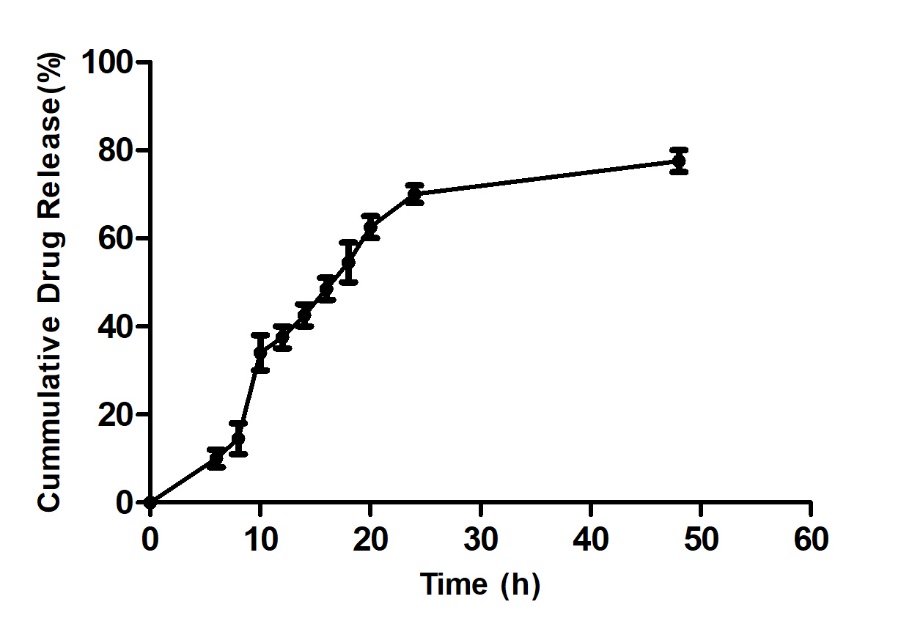


**Figure S1: Release Profile of optimized Inhaled formulation (n=3)**


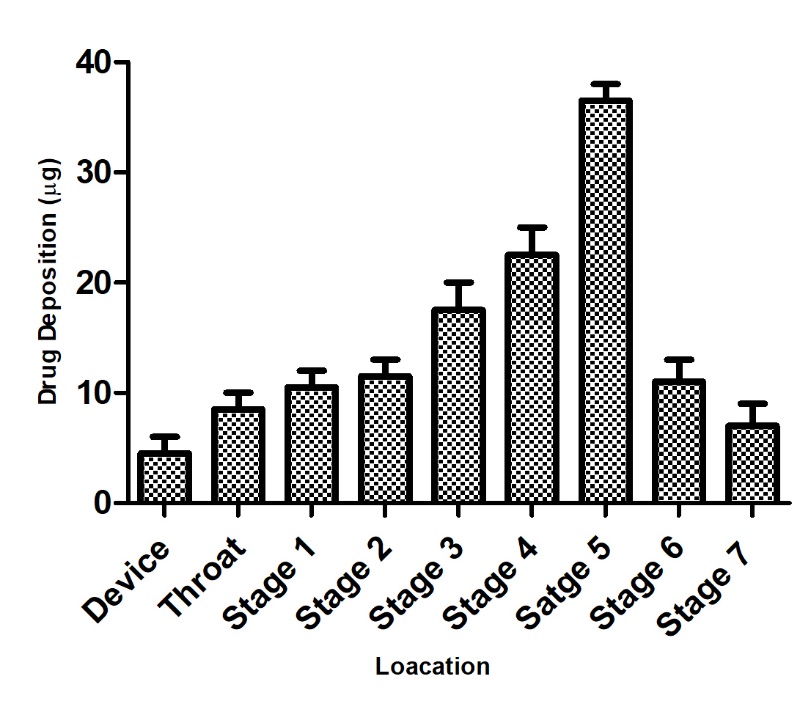


**Figure S2: Deposition of particles in each stage of cascade impactor (n = 3)**

**Table S1: Accelerated Stability Study of Optimized Batch**

| **Time**  **(Month)** | **Particle size**  **(nm)**  **± SD*** | **%Entrapment Efficiency**  **± SD*** | **Percentage**  **drug release**  **in 48 h ± SD*** |
| --- | --- | --- | --- |
| 0 | 273±2.45 | 71.2±1.68 | 79.63±1.89 |
| 3 | 282±1.89 | 70.13±1.32 | 77.25±1.25 |
| 6 | 286±2.01 | 69.89±2.05 | 76.10±1.32 |

*The results are in triplicate (n=3)
