# Supplementary figures and images for: Lung cancer targeting efficiency of Silibinin loaded Poly Caprolactone /Pluronic F68 Inhalable nanoparticles: In vitro and In vivo study
Source: PLoS One. 2022 May 13;17(5):e0267257. doi: 10.1371/journal.pone.0267257 (PMC9106168; doi:10.1371/journal.pone.0267257)

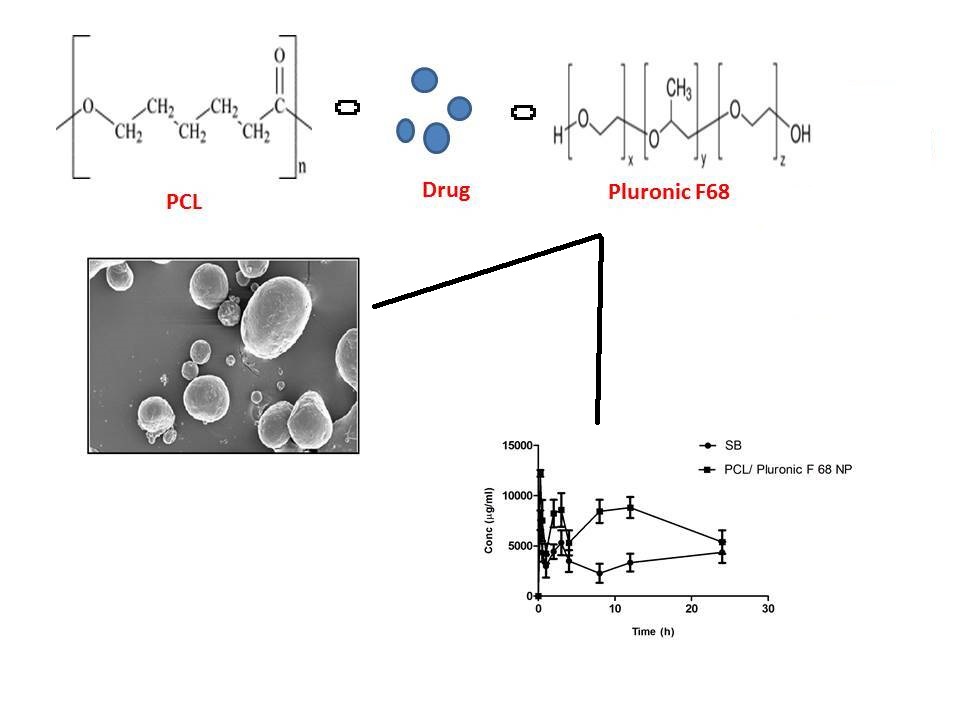

Supplement: S1 Graphical abstract — (JPG) [file pone.0267257.s002.jpg]
